# Functional and Structural Analyses of CYP1B1 Variants Linked to Congenital and Adult-Onset Glaucoma to Investigate the Molecular Basis of These Diseases

**DOI:** 10.1371/journal.pone.0156252

**Published:** 2016-05-31

**Authors:** Antara Banerjee, Subhadip Chakraborty, Abhijit Chakraborty, Saikat Chakrabarti, Kunal Ray

**Affiliations:** 1 Molecular & Human Genetics Division, CSIR-Indian Institute of Chemical Biology, 4, Raja S.C. Mullick Road, Jadavpur, Kolkata-700032, India; 2 Structural Biology and Bio-Informatics Division, CSIR-Indian Institute of Chemical Biology, 4, Raja S.C. Mullick Road, Jadavpur, Kolkata-700032, India; 3 Academy of Scientific & Innovative Research (AcSIR), Central Road Research Institute, Delhi-Mathura Road, New Delhi-110025, India; University of Iowa, UNITED STATES

## Abstract

Glaucoma, the leading cause of irreversible blindness, appears in various forms. Mutations in *CYP1B1* result in primary congenital glaucoma (PCG) by an autosomal recessive mode of inheritance while it acts as a modifier locus for primary open angle glaucoma (POAG). We investigated the molecular basis of the variable phenotypes resulting from the defects in *CYP1B1* by using subclones of 23 *CYP1B1* mutants reported in glaucoma patients, in a cell based system by measuring the dual activity of the enzyme to metabolize both retinol and 17β-estradiol. Most variants linked to POAG showed low steroid metabolism while null or very high retinol metabolism was observed in variants identified in PCG. We examined the translational turnover rates of mutant proteins after the addition of cycloheximide and observed that the levels of enzyme activity mostly corroborated the translational turnover rate. We performed extensive normal mode analysis and molecular-dynamics-simulations-based structural analyses and observed significant variation of fluctuation in certain segmental parts of the mutant proteins, especially at the B-C and F-G loops, which were previously shown to affect the dynamic behavior and ligand entry/exit properties of the cytochrome P450 family of proteins. Our molecular study corroborates the structural analysis, and suggests that the pathologic state of the carrier of *CYP1B1* mutations is determined by the allelic state of the gene. To our knowledge, this is the first attempt to dissect biological activities of CYP1B1 for correlation with congenital and adult onset glaucomas.

## Introduction

Glaucoma represents a group of heterogeneous and complex disorders characterized by a progressive loss of retinal ganglion cells. It is the most common cause of irreversible blindness. According to the latest estimates 5.7 million people are visually impaired and about 3.1 million are blind due to glaucoma worldwide [1]. Among different types of glaucoma, primary congenital glaucoma (PCG) is the most common childhood glaucoma that is observed in the neonatal or infantile periods. This disorder is most likely due to developmental defects in the trabecular meshwork and the anterior chamber angle of the eye. The mode of inheritance of PCG is primarily autosomal recessive with variable penetrance [2]. On the other hand, primary open angle glaucoma (POAG), the most common form of all types of glaucoma, is a complex disease caused by multiple genetic and environmental factors, as well as their interactions. In this disorder, intra-ocular pressure (IOP) usually increases in persons older than 50 years without any subjective symptoms until irreversible damage occurs. However, such an increase in IOP is not a necessary condition for the disease. If the condition is left untreated, it leads to impaired vision and, ultimately, to blindness in approximately 2% of the elderly population [3]. Transmission of the disease occurs mostly in a monogenic form in juvenile onset POAG (JOAG) and in a complex form in adults [4–6].

In 1997, cytochrome P4501B1 (*CYP1B1*; OMIM 601771) was first identified as a causal gene for PCG [7]. Till date mutations in *CYP1B1* are considered the primary cause of PCG in different populations, worldwide [8–10]. For POAG, on the contrary, 16 chromosomal loci have been mapped so far by linkage analysis (GLC1A-P) [11, 12] and at least 20 POAG susceptibility genes have been identified through candidate gene analysis [12, 13]. *CYP1B1* mutations have also been reported to be present in POAG, mostly in the heterozygous state [4, 6, 14, 15]. Initially, *CYP1B1* was suggested to be a modifier gene for the expression of the Myocilin gene (*MYOC*) in patients with JOAG [6]. However, subsequent studies have proposed that *CYP1B1* may play a causative role in JOAG with a possible monogenic association in French, Indian, and Spanish patients [4, 5, 14, 15].

CYP1B1 is a monooxygenase, which catalyzes many reactions involved in the metabolism of endogenous compounds that include 17β-estradiol, retinals, arachidonic acid, and melatonin. The functional implication of *CYP1B1* mutations in differential disease pathogenesis, especially in the causation of PCG and POAG is not well studied. However, it is known that retinoic acid (RA) is critical for ocular development, and the role of CYP1B1 in metabolizing RA might have an implication in the causation of PCG [16, 17]. A recent report from our group has also shown that dysfunction in the 17β estradiol metabolizing activity of some mutants of CYP1B1 can cause MYOC upregulation, which may lead to POAG pathogenesis [18].

In the current cell-based study, therefore, we examine the dual activity of CYP1B1 (retinol and 17β-estradiol metabolism) using natural mutations occurring in glaucoma patients, and we attempt to correlate the differential activity of the enzyme with the clinical phenotypes of the patients (PCG vs POAG) carrying those mutations. We have also performed exhaustive molecular docking, normal mode analysis (NMA) and molecular dynamics (MD) simulation analyses to understand the structural and functional modifications in various naturally occurring CYP1B1 mutations that lead to clinical phenotypes. The active site of CYP1B1 protein, as well as its other family members, is located deep within the protein, close to the heme cofactor [19]. Earlier studies [19–21] have demonstrated that substrate channels or tunnels influence the kinetics of P450 family enzymes and conformational dynamics plays a crucial role in determining the entry and exit of ligands to and from the active site. Crystallography and MD simulation studies on other P450 structures demonstrated the importance of the B-C loop and F-G helix in determining active site access to the substrate [20]. In this study, we have investigated the tunnel properties of the PCG and POAG mutants, and their possible effects on substrate binding. An earlier MD-based study [21] demonstrated the potential effects of only PCG causing mutations on a CYP1B1 homology model. However, in this article, we attempt to dissect the biological activities of the protein through experimentation and dynamics-based structural studies in order to draw correlations with both the congenital (PCG) and adult onset glaucoma (POAG) pathogeneses.

## Materials and Methods

### Selection of *CYP1B1* variants reported in PCG and POAG patients

In this study 23 *CYP1B1* missense variants were selected on the basis of (i) higher frequency of occurrence, and (ii) a large change in the chemical nature (e.g. charge, polarity, molecular size of the side chain etc.) of the mutated amino acid. The selected mutants reported in PCG were mostly in homozygous or compound heterozygous condition whereas in POAG those were mostly in heterozygous condition.

### *CYP1B1* variant clone preparation

A *CYP1B1* true ORF clone was procured from OriGene Technologies, Rochville, USA with a myc epitope tagged at the C-terminal. The 23 mutated clones were created by the QuikChange II Mutagenesis Kit (Agilent Technologies, Santa Clara, CA, USA) using primers specific to the gene. The mutated plasmids were verified by Sanger sequencing. Additional details of primers are available in S1 Table.

### Mammalian cell culture & transfection

HEK293T, and Human Trabecular Meshwork (TM) cell lines were maintained in DMEM (Dulbecco’s modified Eagle Medium, GIBCO BRL Waltham, MA, USA) at pH 7.4 but the media used for the TM cell line contained low glucose concentration (1 g/L). The identity of the TM cell line was confirmed by upregulation of endogenous myocilin by dexamethasone treatment as described previously [18]. The HEK293T cell line from ATCC was selected for mammalian cell-culture experiments because they express almost no endogenous CYP1B1 as determined by Western blot analysis (S1 Fig). The cells were grown in appropriate volume of 10% FBS in high glucose DMEM (GIBCO BRL, Waltham, MA, USA), supplemented with nonessential amino acids (Life Technologies, Carlsbad, CA, USA), L-GlutaMAX, 1% penicillin/streptomycin, and sodium pyruvate. All cultures were grown in a 37°C incubator with 5% CO_2_ under normal humidity.

6 X10^5^ and 3X10^5^ cells were plated on a 6 well plate and 12 well plate respectively in 10% FBS in DMEM. Transient transfections were done with various plasmids (variant clones of *CYP1B1* generated through SDM) and Retinoic Acid Receptor Element (RARE) constructs, procured from SA-Biosciences (QIAGEN, Hilden, Germany), using Lipofectamine 2000 reagent (Invitrogen, Waltham, MA, USA) according to the manufacturer’s protocol. Cells were grown in DMEM serum free medium (Life Technologies, Carlsbad, CA, USA) for 6h, and then shifted to complete growth medium.

### Determination of estradiol metabolism activity of wild type (WT) and variant CYP1B1

Estradiol metabolism activity of the WT and mutants were measured with the CYP450-GLO^™^ Assay kit (Promega, Madison, WI) using the protocol described previously [18] and the estimation of the enzyme activities were done as mentioned below:

First, background luminescence (no cell control) was subtracted from all test samples (WT and all other variants) including the empty vector transfected group.

Second, the values observed for empty vector transfected cells were subtracted from values observed for WT and other mutants. In this way the endogenous activity of CYP1B1 was taken care of.

The enzyme activities of the mutant proteins were expressed as a percentage of the activity retained as compared to the WT protein. Each assay was performed in triplicate and repeated three times, and statistical significance was calculated using Student’s t-test.

### Determination of retinol metabolism activity of wild type and variant CYP1B1

To assess the retinol metabolizing activity of the CYP1B1 variants we designed an indirect strategy. We took advantage of the ability of CYP1B1 to convert retinol into RA. We performed sequential transient transfection of CYP1B1 constructs in HEK293T cell line followed by co-tranfection of an inducible RARE-responsive firefly luciferase construct and another construct constitutively expressing *Renilla* luciferase (SA-Biosciences, Qiagen, Germany). Cells expressing WT and mutant CYP1B1 proteins could then convert retinol into RA, which would eventually bind to inducible-RARE. The method is described in details.

Cells were transiently transfected with WT and variant clones of *CYP1B1*. After 12h RARE constructs (SA-Biosciences, QIAGEN, Hilden, Germany) were transfected in the cell line using Lipofectamine 2000 reagent (Invitrogen) according to the manufacturer’s protocol; 16h post transfection, retinol solution (2μM final concentration), procured from Sigma (St. Louis, MO), was added after media change to each well (according to manufacturer protocol). The cells were incubated for 6h.

Post retinol treated cells were washed with phosphate buffer saline (PBS) and subsequently lysed with luciferase cell culture lysis buffer supplied with the Dual Luciferase Reporter Assay kit (Promega, Madison, WI). After a short vortex, whole cell lysates were centrifuged at 4°C at 13,000 rpm (HERMLE Z 233 MK-2) for 1min and 10μl of the supernatants was mixed with 25μl of luciferase assay reagent-I, Firefly (FL) luminescence was measured as relative luciferase units (RLU) in a GLOMAX luminometer (Promega, Madison, WI). Next, 25μl of Stop & Glo solution was added to stop the reaction and *Renilla* Luciferase (RL) luminescence was measured. For quantifying retinol metabolism activity, the calculation method provided by the manufacturer Cignal RARE Reporter Assay Kit (LUC), QIAGEN, Germany was followed.

First, the FL-RLU value of the negative control (a mixture of the noninducible firefly luciferase construct and the constitutively expressing *Renilla* luciferase construct provided in the kit) was subtracted from the value of all test samples (WT and all other variants) including the empty vector transfected group. Second, the luminescence value of the empty vector transfected group was subtracted from WT and other variants. Third, the FL-RLU value for each variant was divided by the corresponding RL-RLU value to normalize for transfection efficiency. The enzyme activity of the mutant proteins was expressed as percentage of the activity retained as compared to the WT protein. Each assay was performed in triplicate and repeated three times, and statistical significance was calculated using a Student’s t-test.

### Determination of CYP1B1 variant protein turnover rate after addition of cycloheximide (CHX)

Post transfection (14h), 10 μg/ml (final concentration) CHX solution was added in each well. Treated cells were incubated for 12h; protein lysates were prepared at 4h intervals and analyzed through Western blotting techniques. We optimized the concentration of CHX using WT variant before we used it in assay. For this purpose, we assayed the effect of 5, 10, and 15 μg/ml concentrations of CHX on WT expression level at 4, 8 and 12h (S2 Table). We selected a 10 μg/ml dose for our further studies as we intended to show changes in protein level in mutants compared to WT after the addition of CHX.

### Western blotting from mammalian cells

Cells were lysed using NP-40 lysis buffer (150mM Tris-Cl, 50mM EDTA, pH 8.0, 1% NP40) supplemented with protease inhibitor cocktail (1μl/10^6^ cell; Sigma, St. Louis, MO) and was sonicated in a water bath for 5min. Total protein was estimated using Bradford assay (BioRad, Herucles, CA, USA).

Approximately 20μg of total protein was separated on 10% SDS-polyacrylamide (MiniPROTEAN III; BioRad, Herucles, CA, USA) and transferred onto a PVDF membrane (Hybond-P; GE Healthcare, Bedford, UK) by electro blotting using the ECL semi-dry transfer unit (Amersham Biociences, Piscataway, NJ, USA). Membranes were then blocked in 5% BSA in TBS for 2h at room temperature and incubated with the respective primary antibody anti-Myc polyclonal antibody for checking CYP1B1 overexpression (1:1000) (Cell Signaling Technology, Danvers, MA,USA), anti-β-actin (1:2000) antibody (Sigma-Aldrich, St. Louis, MO, USA)] overnight at 4°C. β-actin was used as the loading control. The membranes were washed thrice with TBST 25mM Tris-HCl (pH 7.5), 150mM NaCl, 0.05% tween-20] at 10min interval followed by incubation with the appropriate secondary antibody conjugated with HRP Anti-rabbit (1:60,000) and anti-mouse (1:2000)] (Bangalore Genei, India) for 2h at room temperature. The secondary antibody was washed thrice with TBST followed by two washes with TBS. The ECL-western blotting detection kit (Pierce, Waltham, MA, USA) was used for detection of chemiluminescence. Densitometry for protein band quantitation was performed on scanned films using Image J software on triplicated independent experiments. OD values for each mutants was normalized to OD values of the respective β-actin band. The results were compared using a Student’s *t*-test.

### *In silico* CYP1B1 mutant model generation and molecular docking analysis

The crystal structure of the human CYP1B1 protein (PDB ID: 3PM0) [22] was used for structural analysis. The mutant models of CYP1B1 were generated using the USFC Chimera v1.5.3 structure editing package [23]. Docking of retinol and 17β-estradiol to WT and mutant CYP1B1 structures was performed using the GOLD v5.0.1 [24] package (please see S1 Method for details about the docking protocol).

### Normal mode, molecular dynamics simulation and tunnel analysis

#### Normal mode analysis

The NMA package of the Bio3D [25] program was used to analyze the dynamic behavior of wild type and mutant CYP1B1 structures. The average fluctuations of the 7^th^, 8^th^ and 9^th^ modes were used to compare the systems.

#### Molecular dynamics simulation protocol

CYP1B1 monomer coordinates were collected from the crystal structure of human cytochrome P4501B1 (PDB ID: 3PM0; chain A) [22] as starting structure for initiating the molecular dynamics (MD) simulations. All simulations were performed using GROMACS v4.5.3 [26]. The united atom force field GROMOS96 53a6 [27] was used to describe protein, water and ions. The GROMACS utility, pdb2gmx, was used to rebuild hydrogen positions in the starting structure after ignoring the initial hydrogens in the crystal structure. Both N- and C- termini of the protein were assigned neutral charges and the side-chain functional groups of all the ionizable amino acids were allowed to acquire default ionization states as detected and defined by the pdb2gmx module. The protein structure was placed at the center of the cubic box and solvated with the SPC water model [28]. Periodic boundary condition was applied to minimize edge effects in the solvated structure. The simulation protocol involved energy minimization of the system using steepest descent followed by conjugate gradient algorithms. Next equilibration dynamics of the whole system was carried out for 1ns; ensuring the least disturbance to the starting structure. A leap-frog algorithm was used to integrate Newton’s equations of motion with an integration time-step of 2fs. Twin-range cut-offs of 9Å and 14Å were applied to calculate the van der Waals interactions and the neighbor list was updated every five steps. To compensate for truncation effects, long-range dispersion correction was applied for energy and pressure. The electrostatics were calculated using the Particle Mesh Ewald [29] method. System neutrality was achieved by adding six Na+ ions as counter ions by replacing random water molecules. The LINCS [30] and SETTLE [31] algorithms were invoked to constrain hydrogen bonds and water molecules, respectively. The production run was performed with an NPT ensemble (at physiological temperature, T = 303°K and pressure, P = 1 bar). The system, comprising of the whole protein and the solvent, was separately coupled to the temperature bath to ensure minimum fluctuations in the simulation temperature. During the production run Nosé-Hoover [32, 33] thermostat with temperature coupling, T_T_ = 0.5ps, and Parrinello-Rahman [34] barostat with pressure coupling, T_P_ = 1.0ps, were used. Each CYP1B1 WT and mutant protein was undergone for a production simulation run of 100ns using five nodes (each having six Intel Zeon X5675 processors) on average of a high performance computer (HPC) clusters.

#### MD trajectory, tunnel and essential dynamics analysis

The emergent trajectories were analyzed by employing the inbuilt tools of GROMACS v4.5.3 [26]. Chimera v1.5.3 [23] was used for visualization and image creation. Tunnel analysis was done using CAVER 3.0 program [35] and in-house Perl programs.

MD simulations is used to study the structural motions of proteins while essential dynamics based approach [36] allows to obtain a concise interpretation of the protein motions from the large amount of simulation data. Essential dynamics based approach uses principal component analysis (PCA) to removes the linear correlations among the atomic coordinates of a protein structure and can be described by principal components or PCs to represent a system's motions. These individual PCs represent a set of collective motions exhibited by the 3D structure. Principal components are sorted decreasingly in representing the collective motions, e.g. principal component 1 or PC1 will represent the largest set of collective motions of the system followed by PC2 and PC3. In this study we have used structural ensemble of wild type and mutant CYP1B1 structures obtained from the MD simulation to infer the essential dynamics of the system (please see S1 Method for details about the ED analysis).

## Results

### Selection of mutations

More than 180 different CYP1B1 mutations are linked to different eye diseases including PCG and POAG [9, 10], spread across the entire protein, without any clustering of mutations for a specific disorder (S2A Fig).

For this study, 23 missense mutations were selected belonging to 3 different categories depending on their reported roles in glaucoma pathogenesis; (a) 8 variants were reported exclusively for PCG; (b) 6 exclusively for POAG; and (c) 9 reported for both PCG and POAG (Table 1 and S2B Fig). The mutant proteins resulting from these variants were examined for their biological activity with respect to retinoic acid and 17β-estradiol metabolism.

**Table 1:** Genotype to Phenotype analysis for CYP1B1 variants implicated in POAG and PCG.

MutationStudied [ref.]^1^	Disease (Genotype reported)^2^			2^nd^ mutation reported (if any)^3^	Enzyme Activity for mutant allele studied(% WT)^4^		Inferred Enzyme Activity for Reported Genotype (% WT)^5^		Genotype to Phenotype correlation (Yes/No)^6^
	PCG	POAG	JOAG		Retinol	Steroid	Retinol	Steroid	
p. P52L[42],[43]					0.00 ± 0.00	70.97 ± 4.36			PCG: No;
	Hetero		Hetero				50	85	JOAG: No
p.W57C[44, 45],[4]	Homo	-			221.71±16.19	337.84 ± 20.38	221	338	PCG: Yes;
			Hetero				161	219	JOAG: Yes
p. G61E[44, 46],[15, 43], [47–51]	Homo		Homo		0.00 ± 0.00	0.00 ± 0.00	0	0	PCG: Yes;
		Hetero	Hetero	R355fsX69 in PCG			50	50	POAG: Yes;
	Comp. Het.	Comp. Het		p.Y81N* in POAG			0 for both	5for Y81N; 0 for fs	JOAG:Yes for Homo, if escaped PCG due to incomplete penetrance
p. R117W[52]		-	-		0.00 ± 0.00	0.00 ± 0.00			PCG: No
	Comp. Het.			R469W*			62	3	
p. R117P[53, 54]		-	-		0.00 ± 0.00	70.63 ± 2.60			PCG: Inconclusive (Biochem assay not done for R390H)
	Comp. Het.			R390H			N/A	N/A	
p. M132R[55–57]	Homo	-	-		0.00 ± 0.00	35.35 ± 2.03	0	36	PCG: Yes
p. E229K[10, 42, 45, 53, 54, 58–65],[15, 43],[4, 14]					137.28 ± 6.51	167.65 ± 34.37			PCG: Nofor Comp Het with del mutation; Inconclusive with P193L (Biochem assay not done);
	Hetero	Hetero	Hetero				119	134	POAG: Inconclusive; JOAG: No
	Comp. Het.			P193L, c.1064-1076del			69 for del	84 for del	
p. F261L[42, 66]		-	-		243.15±15.48	22.22 ± 1.86			PCG: Yes
	Comp. Het.			R355fsX69			122	11	
p. D291G[67, 68]	Homo	-	-		0.00 ± 0.00	41.12 ± 18.19	0	42	PCG: Yes
p. G329S[69]					0.00 ± 0.00	0.00 ± 0.00			PCG: Yes
	Comp. Het.			p.T325SfsX104			0	0	
p. R368H[2, 42, 52, 57, 61, 68, 70],[43, 71],[5, 6, 48, 72]	Homo				0.00 ± 0.00	21.29 ± 2.40	0	22	PCG: Yes;
		Hetero		M292K * in POAG			50	61	POAG: Yes for Comp. Het;
		Comp. Het.	Comp. Het.	1546dup10 frameshift in JOAG			90for M292K; 0 for dup/fs	11 for M292K; 11 for dup/fs	JOAG: Likely if accompanied by*MYOC*
p. E387K[8, 58, 63, 73–77],[14]	Homo	-			0.00 ± 0.00	0.00 ± 0.00	0	0	PCG: Yes. Inconclusive for Comp Het with P437L(Biochem assay not done);
				8182delG,268delSNF,P437L in PCG					JOAG: Inconclusive (Biochem assay not done for G232R)
	Comp. Het.		Comp. Het.	G232R in JOAG			0for del	0 for del	
p.R444Q[69, 76, 78, 79]	Homo	-	-		274.15 ± 5.80	25.71± 2.65	274	26	PCG: Yes
	Comp. Het.			3964delC			137	13	
p. R469W[44, 68, 80, 81]	Homo	-	-		124.20 ±25.30	5.79 ± 1.04	124	6	PCG:Yes
p. S28W[15]	-		-		0.00 ± 0.00	600.41 ± 28.58			POAG:.Yes
		Hetero					50	350	
p. Y81N[42],[15],[14],[43]				G61E*	0.00 ± 0.00	10.41 ± 5,50			PCG: No;
	Hetero	Hetero	Hetero				50	55	POAG: Yes;
		Comp. Het.					0	5	JOAG: Yes if accompanied by *MYOC* mutation.
p. Q144H[15]	-		-		82.67 ± 6.36	14.99 ± 2.08			POAG: Yes
		Hetero					91	57	
p. Q144R[82],[51]	NA		-		0.00 ± 0.00	84.80 ± 1.45	0	85	PCG: Predicted for a homozygote;
		Hetero					50	92	POAG: No
p. M292K[72]	-		-		181.50 ± 6.84	0.00 ± 0.00			POAG: Yes
		Hetero					141	50	
		Comp. Het.		R368H*			90	11	
p. V409F[15]	-		-		71.50 ± 12.55	151.49 ± 2.96			POAG: Inconclusive
		Hetero					86	126	
p. F445C[82],[51]	NA		-		226.67 ± 14.53	55.03 ± 1.73	227	55	PCG: Predicted for a homozygote;
		Hetero					164	78	POAG: Inconclusive
p. R523T[4]	-		Homo		210.85 ± 9.93	8.21 ± 7.62	211	8	JOAG: Yes with potentially incomplete penetrance of PCG.
p. D530G[4]	-		-		0.00 ± 0.00	88.19 ± 2.60			POAG: No
		Hetero					50	94	

^**1,**^ Column 1: Numbers in square brackets represent references to the publications reporting the cited study.

^**2,**^ Column 2: Abbreviations: NA, not available (mutation reported without genotype); Homo, homozygote; Hetero, heterozygote; Comp Het, Compound heterozygote. Top, middle and bottom rows represent homozygous, heterozygous and compound heterozygous genotypes, respectively.

^**3,**^ Column 3: Asterisks indicate that the biochemical assay results for those mutations are available (Please see S3 Table).

^**4,**^ Column 4: Enzyme activities are shown as percent of the value obtained for the wild type allele ± SEM.

^**5,**^ Column 5: Enzyme activities for the reported genotypes have been inferred based on an in vitro assay. Top, middle and bottom cells within column 5 in each row provide the inferred enzyme activities for homozygous, heterozygous and compound heterozygous genotypes, respectively. For homozyotes, the activity for the reported mutation (1^st^ column) was considered. For heterozygotes, along with the activity of the reported mutation (1^st^ column), the activity due to the 2^nd^ allele (wildtype) was taken as 100% for calculation. For Compound Heterozygotes, the activity due to the 2^nd^mutation (if available) was used for calculation. For deletion/frame shift mutations the enzyme activity has been inferred to be null and the available assay results for the 2^nd^mutations (marked by asterisk) are given in S3 Table.

^**6,**^ Column 6: Genotype to phenotype correlation is furnished for only reported genotypes except in two cases (Q144R and F445C) where the published report for PCG described a single mutation without the accompanying genotype. The basis for Genotype to Phenotype (G2P) correlation is furnished below:

***For PCG*:**CYP1B1 mediated PCG occurs in an autosomal recessive mode of inheritance.

Hence genotype to phenotype correlation has been proposed based on a biochemical assay done on retinol metabolism by CYP1B1 variant proteins. Both very low and a large excess of retinoic acid levels interfere with the developmental process. G2P correlation has been proposed based on the inferred enzyme activity for the reported genotypes only, based on our in vitro assay results for both mutations (in case of a homozygote or compound heterozygote). If our assay result was not available for the second mutation, an inference has been drawn for the deletion mutation as null activity, but no correlation was attempted for the missense mutation.

***For POAG*:** POAG is a complex disease, which is mostly caused by the interplay of multiple genes and the environment.

The role of CYP1B1 in the case of POAG has been examined based on the report that a low activity of CYP1B1 would help accumulate intracellular estradiol, resulting in the over expression of myocillin, and might lead to adult onset glaucoma. However, while the molecular basis for the pathogenesis of mutant MYOC is known, the potential for over expression of wild-type MYOC causing glaucoma is not yet shown. On the other hand, a higher level of activity of estradiol metabolism can lead to ROS generation and apoptosis, which could ultimately also lead to POAG pathogenesis.

***For JOAG*:** JOAG is a complex disease caused by the interplay of multiple genes and the environment, and clustering in families is more common than in POAG.

No specific role of CYP1B1 has been described, except that it can cause JOAG by a digenic mode of inheritance along with MYOC mutation. However, on rare occasions, a homozygous CYP1B1 mutation has been reported in JOAG without any evidence for the molecular basis for causality.

For structural studies, 17 out of 23 mutations, located in the resolved regions of the CYP1B1 crystal structure, were taken for NMA and MD analysis [36–39] to evaluate their effects on CYP1B1 structural flexibility. Emphasis was given on the B-C and F-G block regions, as prior studies reflected their crucial roles in regulating structural dynamics and ligand tunnels of cytochrome P450 family members [21, 40, 41].

### Functional analysis of CYP1B1 mutants

#### The majority of CYP1B1 mutants show reduced estradiol metabolizing activity

**Fig 1:**
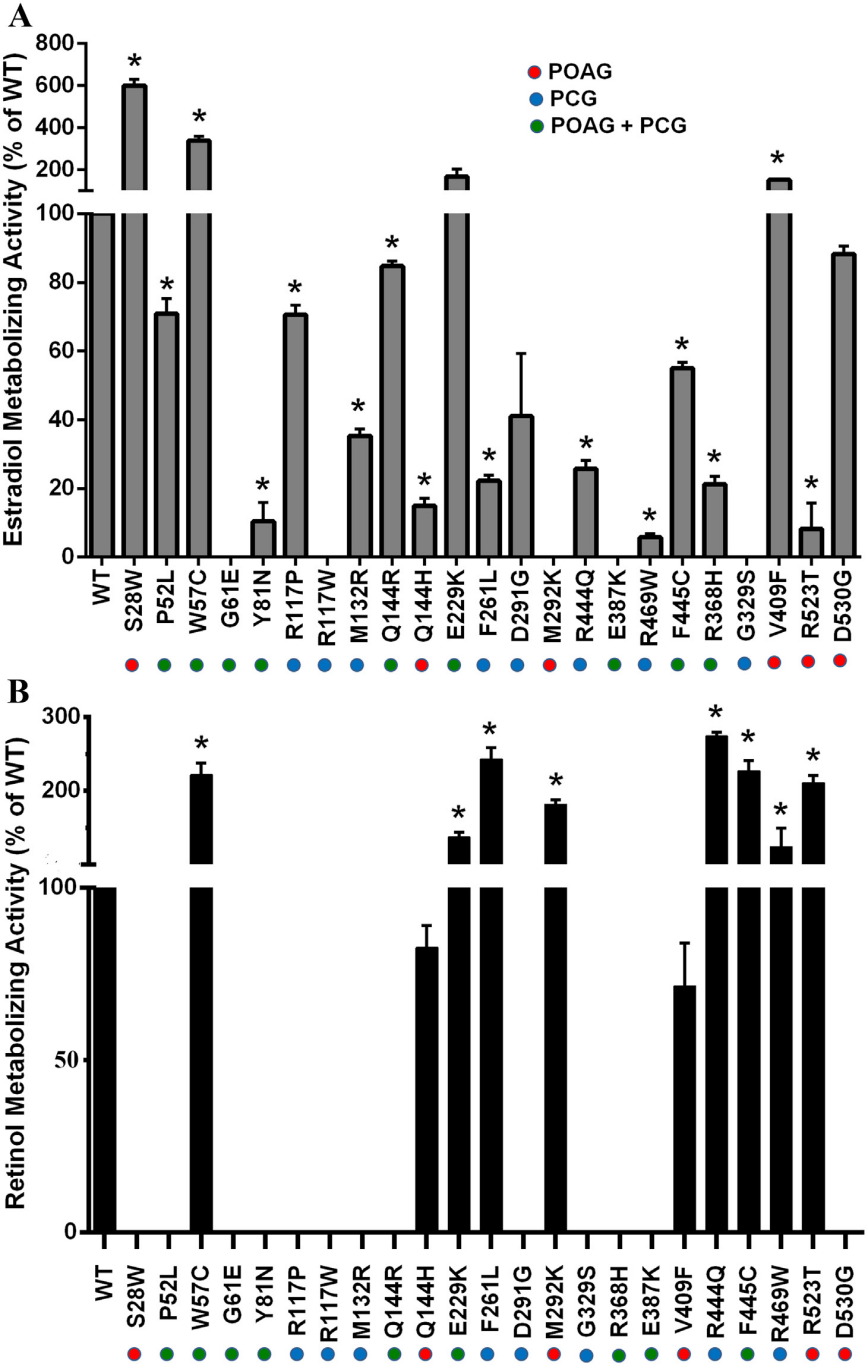
CYP1B1 mutants assessed for 17β estradiol and retinol metabolizing activities. ***Panel A*: *Most CYP1B1 mutants have lower 17β-estradiol metabolizing activity compared to the wild type protein*.** Forty-six hours post-transfection, the steroid metabolism activity of CYP1B1 was measured using the CYP450-GLO^™^ Assay kit. Protein expression of the wild type and mutant CYP1B1 at the time of enzyme assays was estimated by western blot (S3A Fig). Mutations reported to be associated with the type of glaucoma have been indicated by colored circles (POAG: red; PCG: blue; and POAG+PCG: green). ***Panel B*: *CYP1B1 mutants reported in POAG and PCG cases show differential patterns for retinol metabolizing activity*.** To assay retinoic acid metabolism activity, HEK 293T cells were transiently transfected with different CYP1B1 variant clones and CYP1B1 expression was allowed for 12h. Next, cells were transfected with an inducible RARE-responsive firefly luciferase construct mixed with a constitutively expressing *Renilla* luciferase construct available in the SA-Bioscience Kit. After another 16h, retinol was added to each well at 2μM concentration. Six hours post retinol treatment, cells were washed and lysed with luciferase cell lysis buffer. Firefly (FF) and *Renilla* Luciferase (RL) luminescence was measured using the Dual Luciferase kit from Promega. Each assay was performed in technical triplicates and repeated three times. The FF–RLU value was normalized by dividing with RL–RLU value. Cells expressing wild type and mutant CYP1B1 proteins convert retinol into RA, which binds the inducible-RARE construct and luminescence is generated. The enzyme activity of the mutant proteins was expressed as a percentage of the activity retained as compared to the native (wild type) enzyme. Protein expression of the wild type and mutant CYP1B1 at the time of enzyme assays was estimated by western blot (S3B Fig) Data represent the mean ± SEM for a triplicate per group. Data were tested with an unpaired *t* test. Differences in mean values were assessed for statistical significance (*, *p*< 0.01). Experiments were repeated three times.

The enzymatic activities of wild type (WT) and mutant clones were measured to explore whether the mutant CYP1B1 proteins lack 17β-estradiol metabolizing activity, as described previously [18]. Three out of six mutants reported only in POAG (Q144H, M292K, R523T) (Fig 1A) showed significantly reduced estradiol metabolizing activity (0 to 14.99%, p<0.005) with respect to the WT control. The fourth mutant (D530G) also showed reduced enzymatic activity (88.19%±2.60) but the difference was not statistically significant. Interestingly, remaining two mutants (S28W and V409F) in this category showed significantly higher enzymatic activity. S28W showed almost 6-fold (600.41% ± 28.58) and V409F showed 1.5 fold (151.49% ±2.96) higher enzyme activity in comparison to WT. Also, six out of the nine mutants reported for both PCG and POAG (P52L, G61E, Y81N, R368H, E387K, and F445C) was observed to have significantly reduced enzyme activity (0 to 71.09%, p<0.05). Q144R also showed slightly reduced enzyme activity (84.80±1.45). Remaining two mutants, W57C and E229K had almost 3.4 (337.84 ± 20.38) and 1.7 (167.65 ±34.37) fold higher enzyme activity, respectively, in comparison to WT. The mutations reported exclusively in PCG (R117W, R117P, M132R, F261L, D291G, G329S, R444Q, and R469W) were found to have widely varied enzyme activity in the range of null to 70.63% ± 2.60 (Fig 1A).

#### CYP1B1 mutants in general show two discrete patterns for retinol metabolizing activity

In general, either null or significantly higher retinol metabolizing activities were detected for mutants compared to the wild type protein. Our experimental results indicate that mutations that were exclusively reported in PCG patients either completely lacked retinol metabolizing activity (e.g., R117W, R117P, M132R, D291G, and G329S) or showed significantly higher activity e.g. F261L (243.15 ± 15.48%), R444Q (274.15 ± 5.80%), and R469W (124.20 ± 25.30%)] (Fig 1B). Variants reported for both PCG and POAG also followed a similar pattern (Fig 1B). On the other hand, mutants reported solely for POAG patients showed a heterogeneous pattern, i.e. (a) *Null*: 2 variants (S28W, D530G), (b) *Higher than WT*: 2 variants M292K (181.50 ± 6.84%), R523T (210.85 ± 9.93%)] and (c) *Slightly Reduced Activity*: 2 variants Q144H (82.67 ± 6.36%), V409F (71.50 ± 12.55%)] (Fig 1B).

It is worth mentioning here that an optimum level of retinol metabolism is required for normal eye development. Both an excess and a deficiency of vitamin A (retinol) and related compounds (retinoids) has been found to be associated with teratogenesis and malformation of organs including the eyes [16, 17, 83].

#### Correlation of translational turnover rate of CYP1B1 mutants with their enzymatic activities

**Fig 2:**
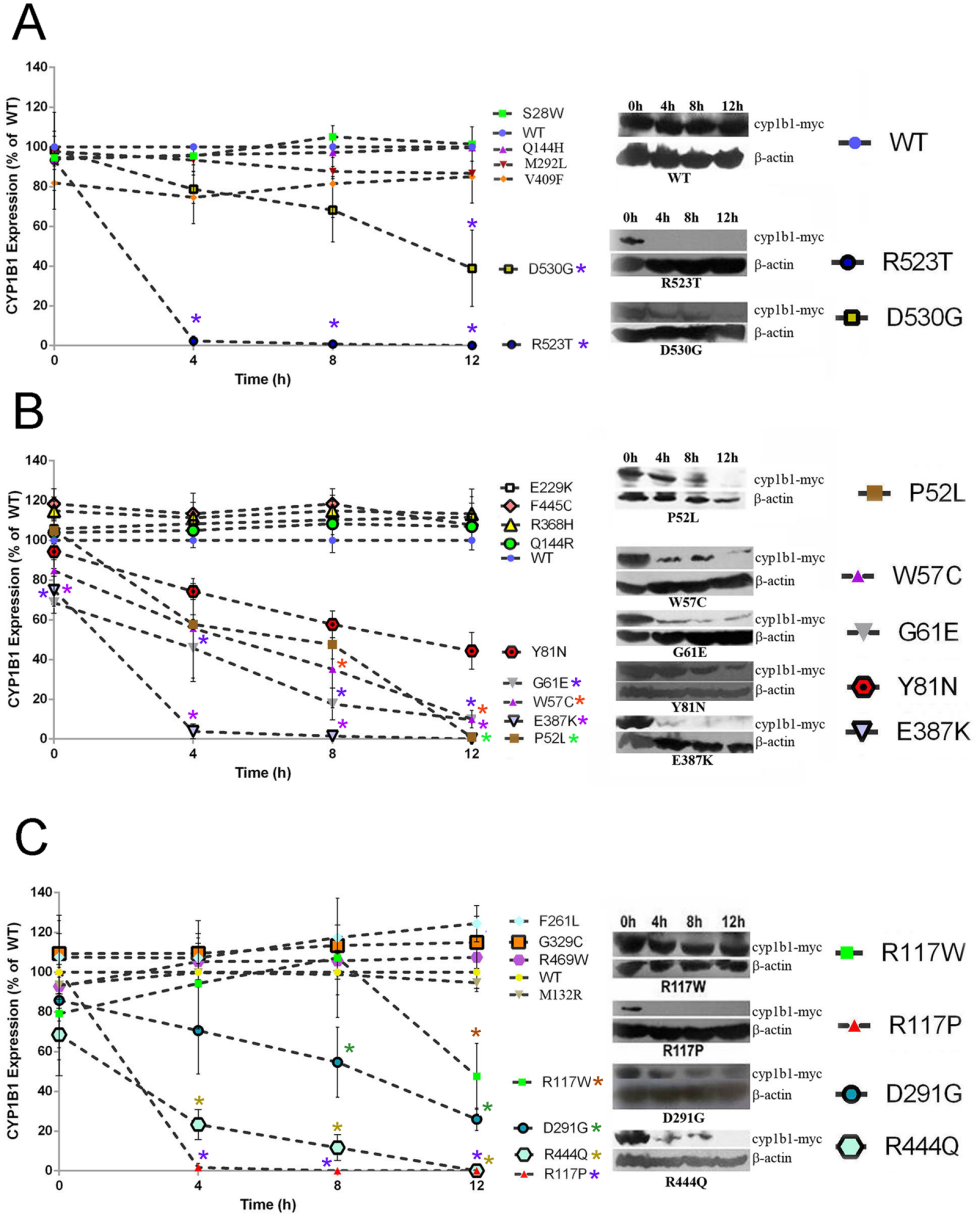
Protein turnover rate of CYP1B1 constructs. Transfected HEK 293T cells were treated with CHX for 12h to inhibit protein synthesis. Twenty μg cell extracts were probed sequentially, by western blot analysis, with appropriate antibodies: Myc (to detect recombinant CYP1B1) Cell Signaling Technology, USA] and β-actin (to serve as a loading control) (Sigma, USA). Immunoblots were scanned, and net pixel intensities of the bands were measured with Image J software. CYP1B1 values were normalized to β-actin, the mean values were taken for three separate transfections, and the relative amounts of CYP1B1 were expressed as a percentage of levels of WT at respective time points. Representative lanes from western blots are furnished on the right side of the panels. ***Panel A*:** Mutations found in only POAG cases; ***Panel B*:** Mutations found in both POAG and PCG cases; ***Panel C*:** Mutations found in only PCG cases. Level of WT protein under similar conditions is shown in panel A. Data represent the mean ± SEM for a triplicate per group. Data were tested by an unpaired *t* test. Differences in mean were assessed for statistical significance (*p*< 0.01). Experiments were repeated three times.

It is suggested that the differential enzyme activity of different CYP1B1 mutants may have correlation with their stabilities after protein expression [42, 84–86]. In this context, we examined the translational turnover rate of the mutant CYP1B1 proteins after addition of CHX and the subsequent effect on their biological activity. It was observed that 12 out of 23 variants did not show any deviation in decay over the time-course (0, 4, 8, and 12h) from that of wild type. Among them, F261L, although showing higher level of expression (124.43%±9.12) at 12h after the addition of CHX, compared to WT, this difference was not statistically significant. The remaining 11 mutants (P52L, W57C, G61E, Y81N, R117P, R117W, D291G, E387K, R444Q, R523T, D530G) were found to be less stable after the addition of CHX (Fig 2). Three of these mutants viz. R117P (reported only in PCG), R523T (reported only in POAG) and E387K (reported both in PCG and POAG) showed little or no expression at 4h after the addition of CHX. For these mutants we estimated the protein levels at time intervals of a shorter duration (15min, 30min, 1h, 2h and 3h) after the addition of CHX (S4 Fig). In case of R117P, the translational arrest started immediately after 30 min of CHX treatment and showed almost no detectable protein level by 3h. E387K and R523T showed relatively greater stability compared to R117P. While the expression of E387K began to decline from 1h after CHX addition, R523T was stable up to 3h (S4 Fig).

Among the 11 mutants with lower stability, it was observed that the instability of seven (P52L, G61E, Y81N, R117W, D291G, E387K, D530G) could result in a reduction in both the estradiol- and RA- metabolizing activities (Fig 1A and 1B), i.e. the unstable nature of these mutant proteins was possibly the cause of their lower enzymatic activities. In contrast, a direct correlation is lacking for the other 4 mutants (W57C, R117P, R444Q, and R523T). In the case of R117P, estradiol metabolism was found to be almost similar to WT (Fig 1A), while in W57C, R444Q and R523T, retinol metabolism was found to be a few folds higher (Fig 1B).

The apparent lack of correlation for these 4 mutants could possibly be due to their different rates of translational turnover over the course of time when assays were done without addition of CHX (S5 Fig). Our results revealed that the increase in level of protein expression of these mutants (W57C, R117P, R444Q, and R523T) was quite low during the initial hours (S5 Fig), which gradually reached almost comparable levels with WT when enzyme assays were done (i.e. 36h for retinol, and 46h for estradiol metabolizing assay). At 14h after transfection (i.e. CHX addition time point), the expression of these mutants was already low, and after inhibiting protein synthesis with CHX the levels decreased further. Thus, if the results for these mutants are viewed along with other mutants, the low level of signal at early time points may appear to be due to low stability for these mutants, which is not supported by following the assay for a longer period without addition of CHX (S5 Fig). Therefore, the lack of correlation of these four mutants with the phenotype might be the limitation of the in vitro assay, resulting from slower translation rates of these mutant proteins.

### Structural analysis of CYP1B1 mutants

Our functional data indicate that mutations reported in PCG patients either completely lacked retinol metabolizing activity or showed significantly higher enzyme activity compared to WT. On the other hand, estradiol metabolism activity was spread over in the range of 36.33% ± 2.09 to 89.53% ± 2.24. First, we were interested in investigating the structural reason behind two distinct functional results observed in the case of retinol metabolism. NMA analysis of only PCG-causing mutants showed a similar trend of flexibility in the B-C and F-G block regions (S2C Fig) with R117P and F261L mutants showing the highest extent of altered flexibilities. We selected these two mutant proteins for further in-silico structural analysis.

In the case of POAG mutants and POAG+PCG cases (S2C Fig), no specific flexibility pattern was observed. However, Q144R, which is involved in both PCG and POAG pathogenesis, and showed flexibility similar to that of only PCG causing mutants, and an exclusive POAG-causing mutation involving the same residue (Q144H) were also selected for further in depth Molecular Dynamic (MD) based structural analysis. Notably, Q144R caused null retinol metabolism and almost normal steroid metabolism, whereas Q144H showed slightly reduced retinol metabolism and significantly reduced steroid metabolism activity.

#### Lower flexibility in crucial loops and gain of ligand tunnel pathway in F261L might cause gain of function in retinol metabolism

**Fig 3:**
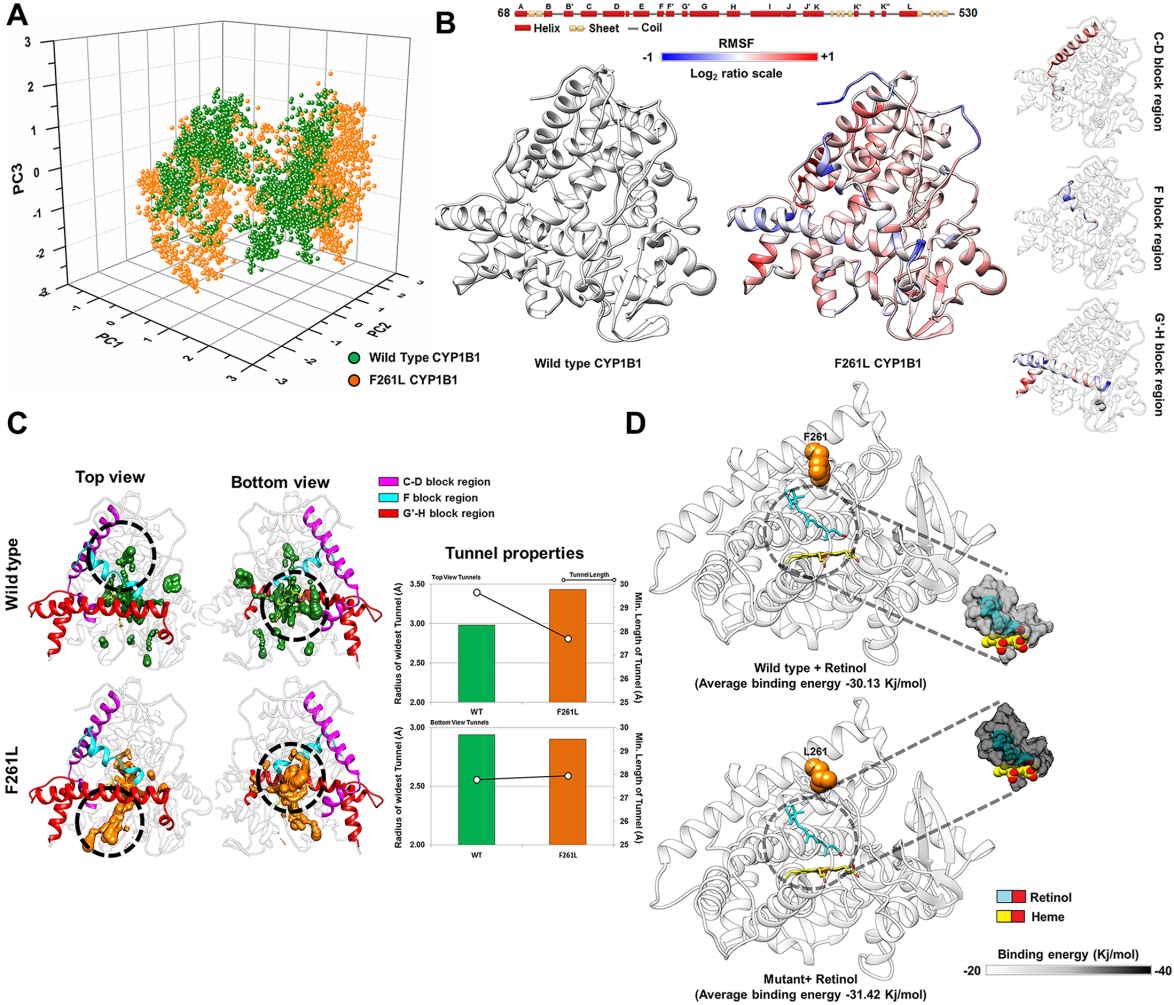
MD simulation analysis of the F261L and wild type CYP1B1 structures. ***Panel A*** shows a similar distribution of the first three major principal components of F261L and wild type (WT) CYP1B1 structures, suggesting relatively unchanged functional motions in the F261L mutant as compared to the WT structure. ***Panel B*** shows the altered flexibility pattern of the F261L mutant as compared to wild type. The log_2_ratio is calculated as log2(RMSF value of ith residue in F261LRMSF value of ith residue in WT). Therefore a positive value indicates an increase in flexibility in the F261L mutant and a negative value indicates a decrease in flexibility as compared to WT CYP1B1. F261L mutant has a significantly altered flexibility pattern within the C-D, F and G'-H block regions, shown separately in the right hand side of the panel. ***Panel C*** shows the altered tunnels in two different orientations (top and bottom view) of the F261L and WT CYP1B1 structures. The upper panel of "Tunnel properties" shows the radius (in **bar plot**) and length (in **black line**) of the tunnels (Top view orientation) in the mutant (orange) and WT (green) structures while the lower panel shows the similar properties of tunnels observed in the bottom view orientation. ***Panel D*** shows docked retinol in the WT and F261L mutant CYP1B1 structures. The panel also shows an overall increase in binding energy in F261L mutant retinol binding observed through MD simulation.

The PCG causing F261L mutant shows higher retinol activity compared to the wild type protein. MD simulations of F261L with retinol showed that the essential dynamics (ED) (please refer to S1 Method for details about the ED analysis) [36–39, 87–91] of the mutant is relatively similar to its WT counterpart (Fig 3A and S6 Fig), suggesting unchanged functional motions between the F261L and WT structures. However, root mean square fluctuation (RMSF) of the C-D, F and G'-H block residues of the F261L protein showed the most altered flexibility compared to WT (Fig 3B and S7A Fig). Earlier studies [19–21] showed that these blocks are important for regulating the dynamics of cytochrome P450 family members and ligand tunnel formation. A mutation within these blocks may influence the structural flexibility as well as ligand tunnel properties. To understand the effect of the mutation on tunnels, we carried out tunnel analysis using the CAVER 3.0 [35] program. Results showed a retinol-specific tunnel in F261L near the F/G region with a shorter length and broader radius (top view of Fig 3C and upper panel of Tunnel properties), while the bottom view tunnels possess similar tunnel properties as that of the WT protein (Fig 3C, lower panel of Tunnel properties). Altered flexibility was also observed to influence the residues surrounding the tunnel (≤ 5Å radius) (S7B Fig). MD simulations showed a lower retinol binding energy profile in the mutant (a 4.2% increase in overall retinol binding energy is observed as compared to WT) (Fig 3D and S7C Fig) and an altered distribution of the Asp, Ile and Leu residues surrounding (≤ 5.0Å) the retinol substrate bound to F261L (S7D Fig) when compared to the WT.

#### Altered structural flexibility in the R117P mutant probably leads to heme instability, causing loss of retinol metabolism activity

**Fig 4:**
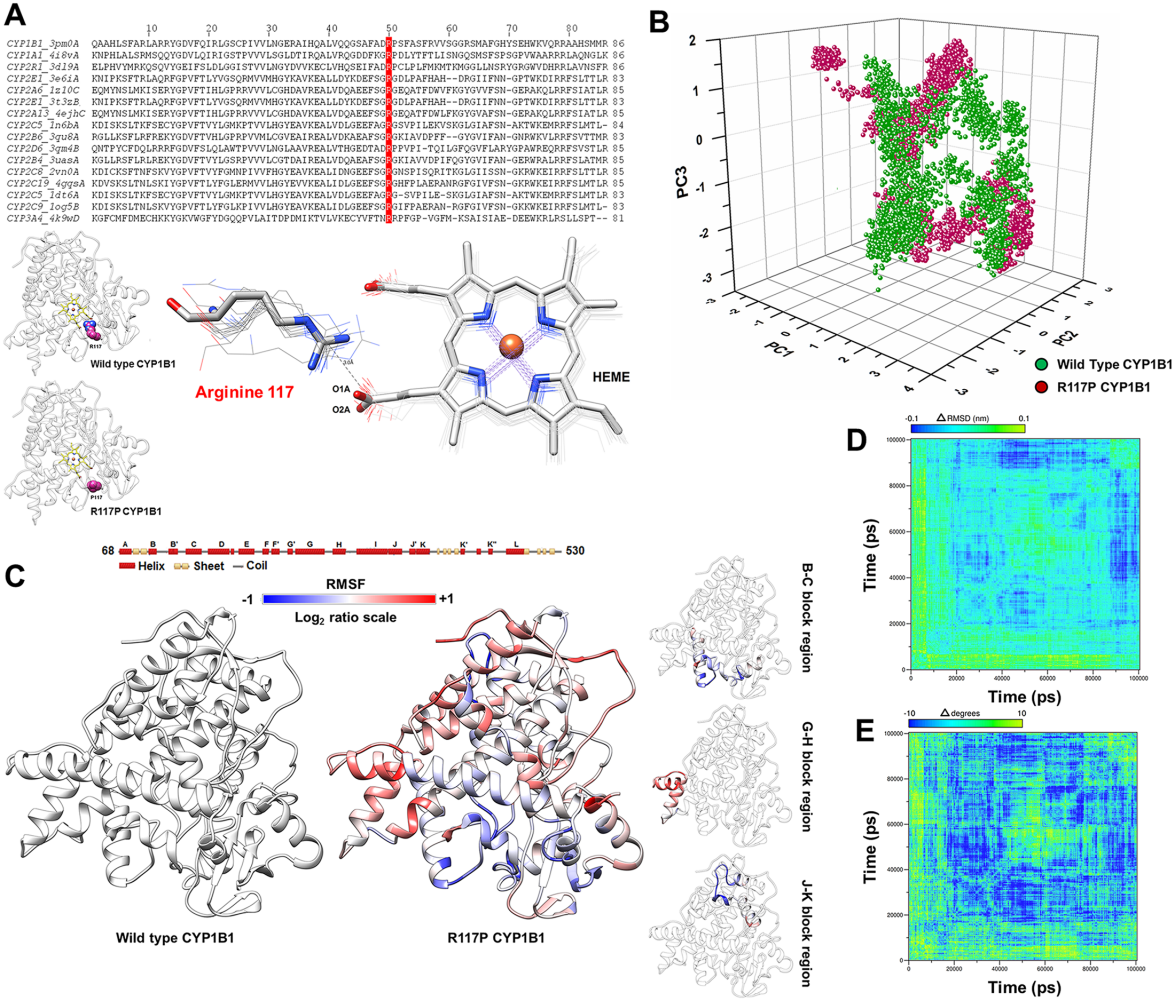
MD simulation analysis of the R117P and wild type CYP1B1 structures. ***Panel A*** shows a multiple sequence alignment of CYP1B1 homologs. The red color indicates the conserved Arginine 117 position in other homologs. Thelower panel shows the structural importance of Arginine 117in their interaction with the O1A/O2A atom of the heme ligand. ***Panel B*** shows a similar distribution of first three major principal components of R117P and wild type (WT) CYP1B1 structures. *Panel B* shows that the R117P mutant possesses a significantly altered flexibility pattern within the B-C, G-H, and J-K block regions. ***Panel C*** shows the altered flexibility pattern in the R117P mutant as compared to WT. The log_2_ ratio is calculated as log2(RMSF value of ith residue in R117PRMSF value of ith residue in WT). Therefore, a positive value indicates an increase in flexibility in the R117P mutant and a negative value indicates a decrease in flexibility as compared to WT CYP1B1. The R117P mutant has a significantly altered flexibility pattern within the B-C, G-H and J-K block regions, shown separately in the right hand side of the panel. ***Panels D* and *E*** illustrate RMSD deviation and average bond angle deviation of the heme ligand in ΔRMSD and Δdegrees matrices, respectively. The difference matrices were calculated by subtracting RMSD and average bond angle values of mutant CYP1B1 from that of WT CYP1B1. The fluctuations and bond angle deviations in the initial stages of the MD simulation indicate a potential instability in the heme ligand binding affinity within the mutant protein.

A sequence and structural analysis of cytochrome P450 proteins showed that R117 of CYP1B1 is absolutely conserved among its family members (Fig 4A, upper panel) and forms a hydrogen bond with the O1A/O2A atom of the heme ligand (Fig 4A, lower panel). Thus in the R117P mutation, the loss of this crucial hydrogen bond interaction with the O1A/O2A atom might cause reduced heme binding stability. The ED distribution (please refer to S1 Method for details) (Fig 4B and S6 Fig) between WT and R117P protein indicates the existence of relatively distant functional motions between the two proteins. However, the RMSF analysis of R117P showed a significant altered fluctuation within the B-C, G-H and J-K regions (Fig 4C and S8A Fig). Contact analysis of heme O1A/O2A atom within 3.5Å of radius (S8B Fig) showed altered interacting residues with the O1A/O2A atoms, probably arising from the change in the mutant protein flexibility. The probable loss of the heme interactions is also visible from the differences in root mean square deviation (RMSD) (Fig 4D) and average bond angle deviation (Fig 4E) matrices of heme, where fluctuations and bond angle deviations in the initial stages of MD simulation indicate a potentially higher instability in the heme of the mutant protein.

#### Altered structural flexibility and ligand tunnel properties in Q144R mutant might cause the loss of retinol metabolism activity

**Fig 5:**
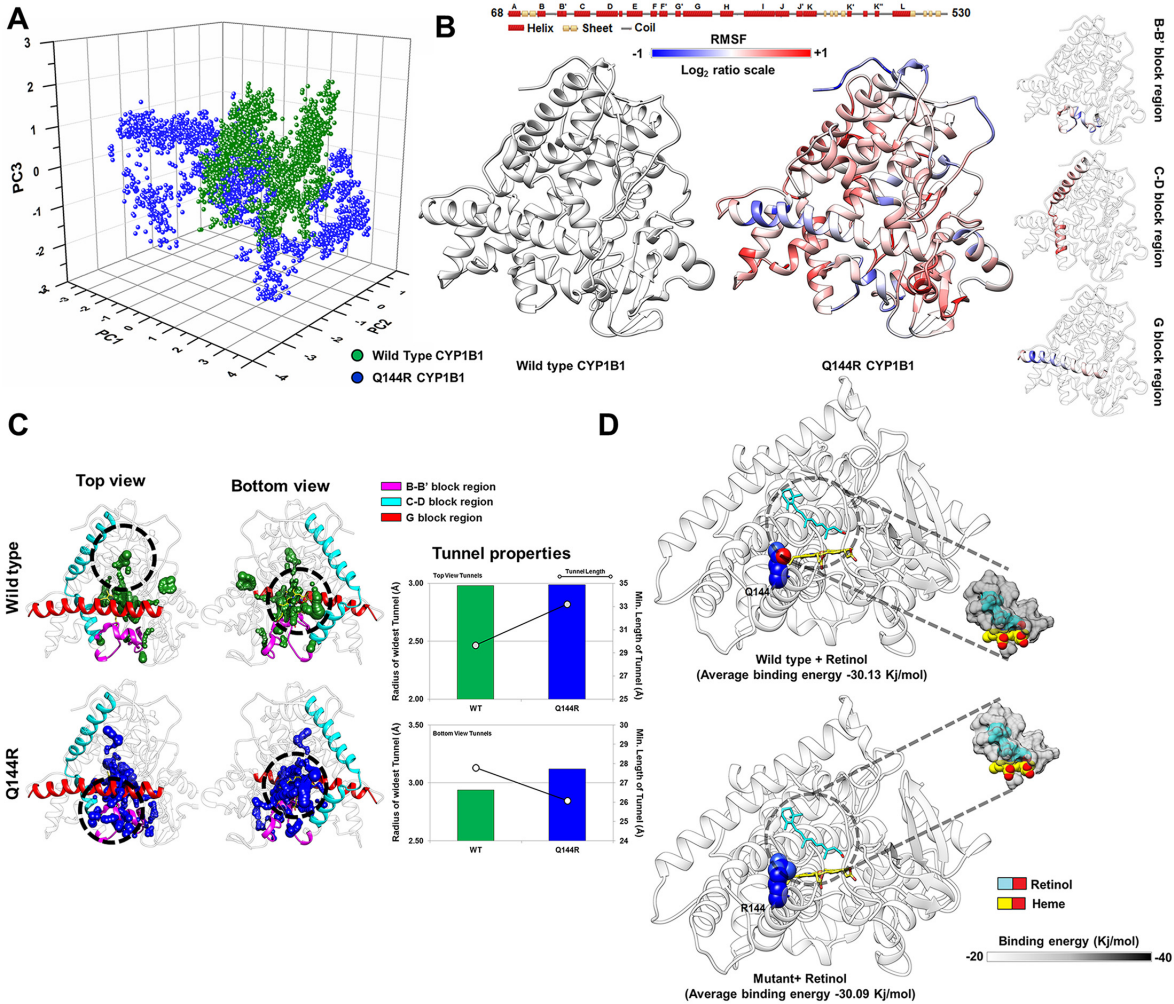
MD simulation analysis of the Q144R and wild type CYP1B1 structure. ***Panel A*** shows the distribution of the first three major principal components of the Q144R and wild type (WT) structures. The distributions are observed to be different, suggesting an altered functional motion in the Q144R mutant. ***Panel B*** shows the altered flexibility pattern of the Q144R mutant as compared to WT. The log_2_ ratio is calculated as log2(RMSF value of ith residue in Q144RRMSF value of ith residue in WT). Therefore a positive value indicates an increase in flexibility in the Q144R mutant and a negative value indicates a decrease in flexibility as compared to WT CYP1B1. The Q144R mutant has a significantly altered flexibility pattern within the C-D, F and G'-H block region, shown separately in the right hand side of the panel. ***Panel C*** shows the altered tunnels in two different orientations (top and bottom view) of the Q144R and WT CYP1B1 structures. The upper panel of "Tunnel properties" shows the radius (in **bar plot**) and length (in **black line**) of the tunnels (top view orientation) in the mutant (blue) and WT (green) structures. The lower panel shows the similar properties of the tunnels observed in the bottom view orientation. ***Panel D*** shows docked retinol in the WT and mutant CYP1B1 structures. The panel also shows an overall decrease in binding energy in retinol binding for the mutant protein, observed through MD simulation.

Q144R CYP1B1 showed a null retinol and almost similar 17β-estradiol activity to WT. It is reported for both PCG and POAG pathogenesis. The difference in ED distribution (please refer to S1 Method for details) observed from MD simulations of WT and Q114R mutant in the presence of retinol can be related to the null activity of Q114R in retinol metabolism (Fig 5A). Comparison of the Q144R RMSF with that of WT (Fig 5B and S9A Fig) showed a decrease in B-C loop, G helix flexibility and an increase in C and D helix flexibility. Significantly altered regions are shown separately in the right hand side of the panel in Fig 5B. Similarly, a comparative tunnel analysis of both the WT and mutant structures showed the presence of an altered retinol-specific tunnel in Q144R (Top view, Fig 5C). The tunnel properties of Q144R showed that the top view tunnels have a similar tunnel radius and a longer length (Fig 5C, upper panel of "Tunnel properties") compared to that of the WT. In bottom view tunnels, a moderate increase in tunnel radius and length (Fig 5C, lower panel of "Tunnel properties) was observed as compared to the WT protein. Tunnel lining residues (≤ 5.0Å) were also found to be different in Q144R as compared to the WT tunnels (S9B Fig). Retinol binding in Q144R showed a slightly higher retinol binding energy compared to the WT structure, especially around 80–100 ns (a 0.13% decrease in overall retinol binding energy is observed in mutant as compared to WT) (Fig 5D and S9C Fig). A contact residue analysis (≤ 5.0Å) of retinol (S9D Fig) showed that the type of residues surrounding the bound retinol is similar in Q144R apart from the presence of a high frequency of Phe and a low frequency of Leu residues as compared to the WT.

#### Lower flexibility in crucial loops and the loss of a ligand tunnel pathway in Q144H probably caused the significantly reduced estradiol metabolism

**Fig 6:**
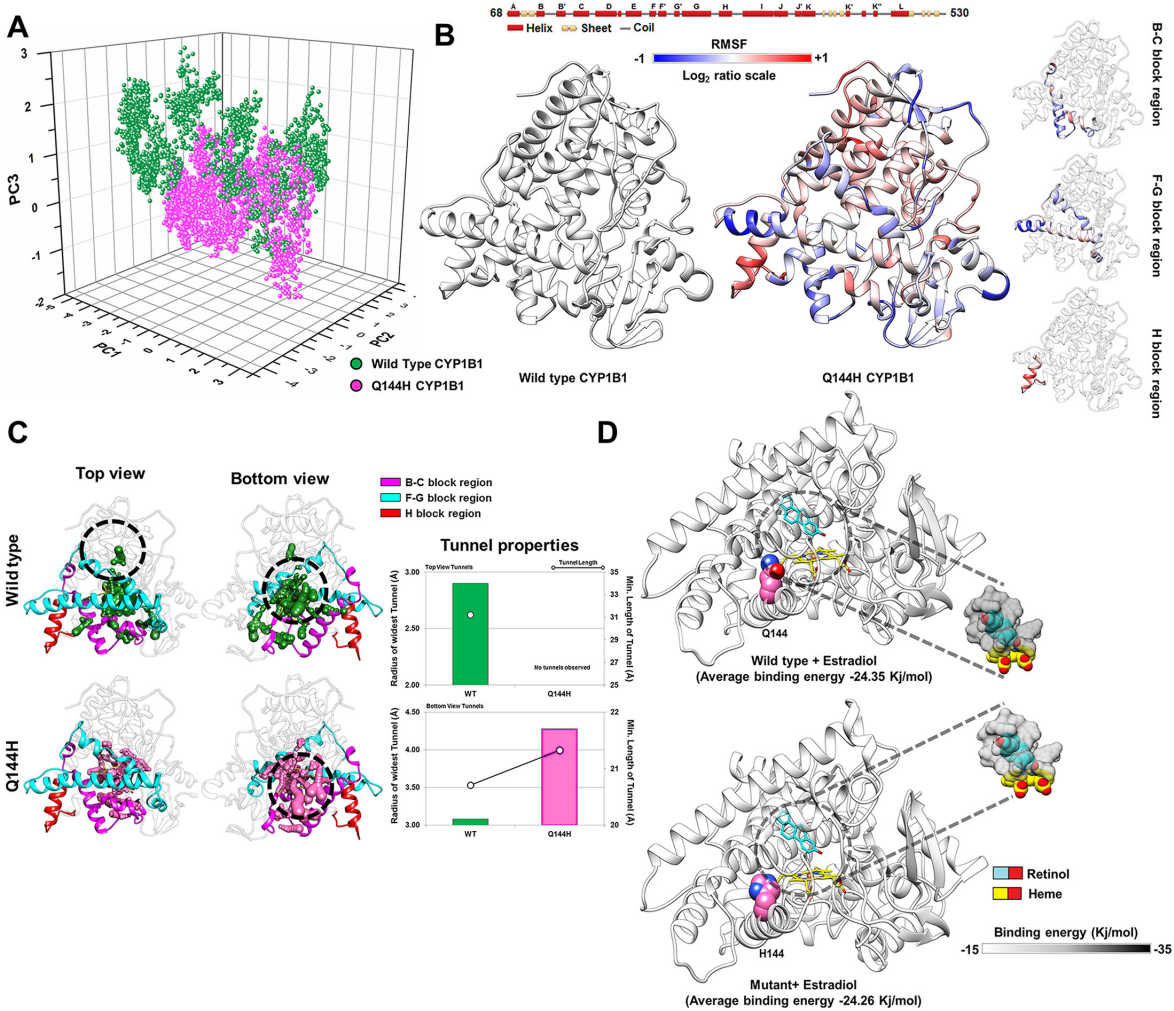
MD simulation analysis of the Q144H and WT CYP1B1 structures. ***Panel A*** shows marginally similar distributions of the first three major principal components of the Q144H and wild type (WT) CYP1B1 structures. ***Panel B*** shows that the Q144H mutant possesses a significantly altered flexibility pattern within the B-C, F-G and H block regions. The log_2_ ratio is calculated as log2(RMSF value of ith residue in Q144HRMSF value of ith residue in WT). Therefore, a positive value indicates an increase in flexibility in the Q144H mutant and a negative value indicates a decrease in flexibility as compared to WT CYP1B1. The significantly altered flexible regions are shown separately in the right hand side of the panel. ***Panel C*** shows the altered tunnels in two different orientations (top and bottom view) of the Q144H and WT CYP1B1 structures. Interestingly no tunnel was observed in the top orientation of the Q144H structure. The upper panel of "Tunnel properties" shows the radius (in **bar plot**) and length (in **black line**) of the tunnels (Top view orientation) in the mutant (pink) and WT (green) structures. The lower panel shows similar properties of tunnels observed in the bottom view orientation. ***Panel D*** shows docked retinol in the WT and mutant CYP1B1 structures. The panel also shows an overall decrease in binding energy in retinol binding for the mutant protein, observed through MD simulation.

Q144H CYP1B1 shows a reduced estradiol activity and is associated with POAG. In this case, MD simulation of 17β-estradiol docked Q144H and WT CYP1B1 structures were performed and subsequent ED analysis (please refer to S1 Method for details) showed a similar distribution of the first three principal components between the Q144H and WT structures (Fig 6A). The B-C and F-G block residues of Q144H showed reduced flexibilities while an increase is observed in H-helix flexibility (Fig 6B and S10A Fig). The tunnel analysis showed the loss of a tunnel in Q144H as compared to the wild type structure (Fig 6C, top view panel) but the bottom view panel (Fig 6C) showed the presence of 17β-estradiol-specific tunnels in both the Q144H and WT structures. However, the tunnels of Q144H in the bottom view region showed better tunnel properties compared to the WT protein (Fig 6D, lower panel of “Tunnel properties”). The overall binding energy of 17β-estradiol in Q144H obtained from MD simulation was slightly decreased as compared to its WT form (a 0.35% decrease in overall retinol binding energy is observed as compared to WT) (Fig 6D and S10B Fig) and the number of hydrogen bonds formed by 17β-estradiol was much lower in Q144H compared to WT (S10C Fig).

### Genotype to phenotype analysis for CYP1B1 variants implicated in POAG and PCG

We hypothesize that the genotype of *CYP1B1* in an individual, activity of the expressed gene product towards its substrate (retinol and estradiol), along with other, yet undescribed, factors finally determine the manifestation of the glaucoma phenotype. Thus, a CYP1B1 variant for which the retinol metabolic activity is critically low, if present as homozygous or compound heterozygous state with a similar metabolic fate, would result in a PCG phenotype. However, a carrier of a single allele of such a functionally deficient variant will not have the disease—as typically expected for an autosomal recessive mode of transmission. Similarly, an individual carrying a CYP1B1 variant that has no effect on retinol activity, but is deficient in metabolizing estradiol, would not have PCG phenotype even with a homozygous genotype, but would be likely to have POAG as an adult.

It is also worthwhile to mention here, as previously described, that a complete lack of and an excess of retinol and retinoids are both associated with the malformation of organs, including the eyes [16, 17, 83]. Hence, a CYP1B1 variant that results in a few fold higher activities relative to normal might also cause PCG depending on its level of expression during development.

Table 1 summarizes the genotypes in PCG and POAG patients, enzymatic activities, inferred CYP1B1 activities, and the possible explanations for disease pathogenesis for each mutation. It was observed that all of the mutants that are reported to be in homozygous or compound heterozygous condition in patients show either very high or null retinol metabolism in the vitro assay (Table 1, Fig 1B). We propose that this distinct alteration in retinol metabolism in individuals bearing these mutants resulted in PCG. On the other hand, mutants appearing in heterozygous condition with low or much higher estradiol metabolism activity mostly leads to the development of POAG.

In such cases, where a single heterozygous mutation in CYP1B1 has been reported in a PCG patient, it is likely that either CYP1B1 is not the underlying causative gene in the patient or the second mutation in the gene is present but not identified. On the other hand, in the case of POAG patients, where the reported CYP1B1 mutations are present in heterozygous condition, a single dose of the WT allele produces necessary threshold amount of RA for normal eye development. If a single defective allele, acquires very high retinol metabolism activity (enough to produce a teratogenic effect) in any patient, it could lead to the precipitation of PCG.

**Fig 7:**
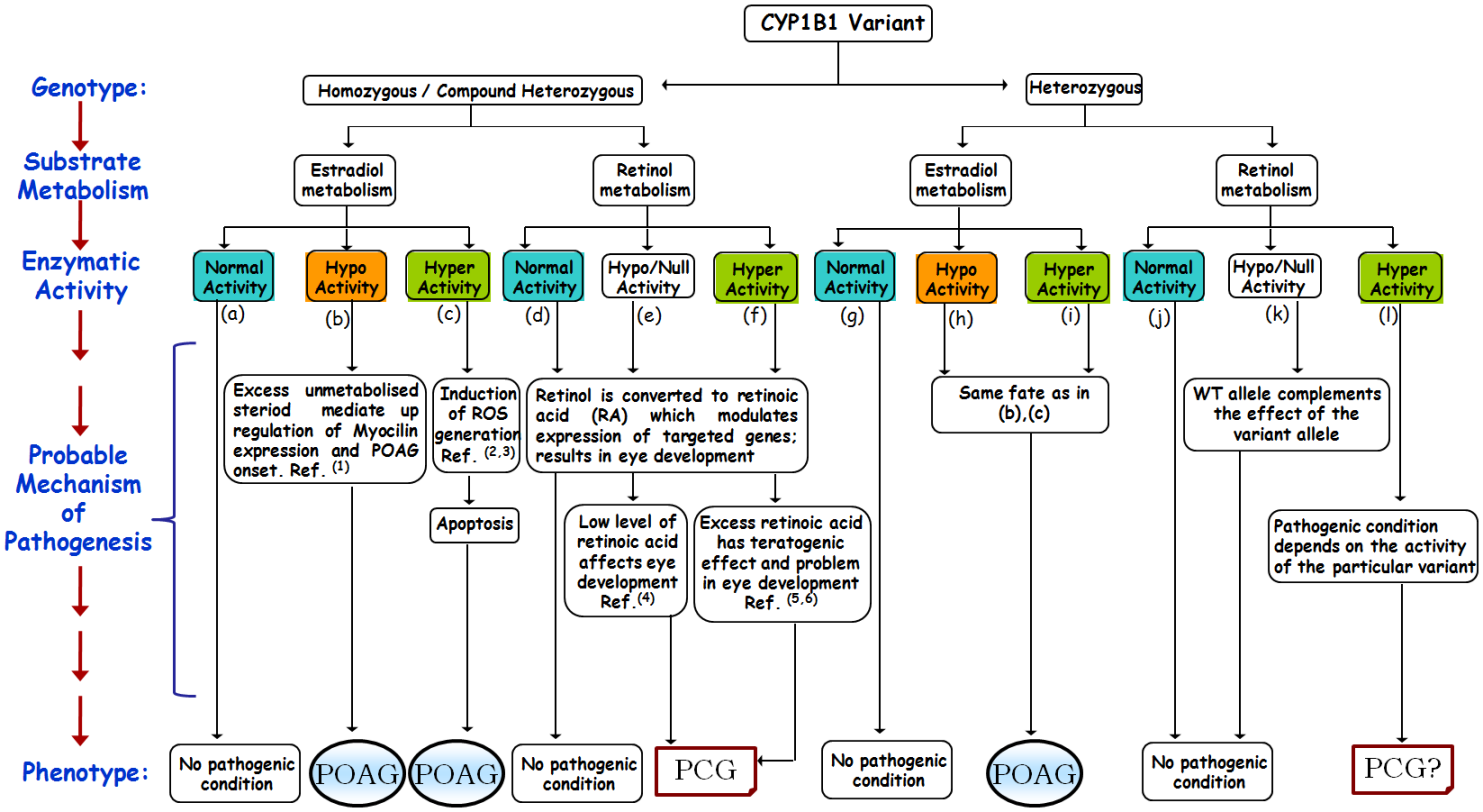
Genotype to phenotype correlation for the role of *CYP1B1* in glaucoma pathogenesis (PCG vs POAG). The flowchart shows the potential activity of CYP1B1 variants for two different substrates (estradiol and retinol), as estimated by an in vitro cell based assay in HEK293T cells and attempted correlation of the biochemical activities (based on genotype) with potential glaucoma pathogenesis. Ref.^(1)^[18]; Ref.^(2,3)^[92, 93]; Ref.^(4)^[17]; Ref.^(5,6)^[17, 83].

A comprehensive understanding of our hypothesis is depicted in the proposed model of disease occurrence (Fig 7).

## Discussion

CYP1B1 is a multifunctional enzyme with diverse substrate specificity, known to be involved in the onset of PCG and POAG. However, the functional role of mutated forms of this enzyme in the different forms of glaucoma is not well understood. In the current study, we attempted to correlate retinol metabolism with glaucoma pathogenesis, especially with PCG, in a cell culture system through an extensive study with 23 CYP1B1 mutations. Notably, it was evident from our genotype to phenotype correlation analysis that dysfunction in one *CYP1B1* allele is sufficient to cause disorders like POAG, as *CYP1B1* mutations occur mostly in heterozygous condition in these diseases, but not in PCG where defects of both the alleles are required for precipitation of the disease phenotype.

Both mouse and human *CYP1B1* orthologs can metabolize retinol and retinal to RA. RA is the ligand for nuclear retinoic acid receptors (RAR) and retinoate X-receptors (RXR). The binding of RA to these receptors results in transcriptional activation of various other genes involved in normal developmental processes. In the present study, we found that *CYP1B1* mutations that are reported only in PCG or both PCG and POAG patients showed either null or over-production of RA. A previous study reporting four mutations in PCG patients (Y81N, E229K, A330F, R368H) showed that retinoid metabolism was severely impaired in the Y81N and R368H mutants, moderately in A330F, and slightly in E229K [84]. Our results with the Y81N and, R368H mutants in the HEK cell line are similar to the previous report, but E229K shows slightly higher activity with respect to WT. The assays done in the previous study is different from ours: The mutant proteins were expressed in bacteria, isolated, and then assayed. In contrast, we have done the experiment in a cell-based system, transfected HEK cells with mutation-specific clones and then estimated the activity in the cell extract. Thus, some differences between the results of the two studies would not be entirely unexpected. Moreover, this result is consistent with the evolutionary conservation of positions Y81 and R368, but not of E229 throughout orthologs and paralogs in different mammalian species, as previously reported [15]. Similarly, it has been reported in an animal study that an optimum retinol metabolism is necessary for normal eye development; deprivation or excess feeding with vitamin A metabolites (RA in particular) to pregnant mammals has been found to be associated with malformation of the eyes [94].

Further, our results showed that most of the POAG mutations caused reduction in CYP1B1 steroid metabolizing activity, providing further credence to the preliminary observation made previously from a much smaller sample size [18]. It is believed that in the case of steroid induced glaucoma, over-expression of myocilin is one of the triggering factors for glaucoma causation; however, the notion is still controversial.

Four mutants, S28W, W57C, E229K and V409F showed higher enzymatic activities relative to the WT control, respectively. A previous report showed that a higher enzymatic activity of CYP1B1 caused accumulation of excess 4-OH-estradiol, leading to formation of quinones. Quinones can create oxidative stress and induce ROS generation, which may cause cell death in TM and retinal ganglion cell and thus could be involved in POAG pathogenesis [92, 93, 95]. Our results for assay for selected mutants are overall similar to the previously reported data with larger variation in a few cases (e.g. P52L, R117W, and E229K). Notably, in three different previous studies [18, 42, 84] E229K showed variable enzyme activity, as also in our present study. This might result from use of different expression vectors, different cell lines, substrate analogues and the method used for assay.

The underlying reasons for the differential activity of the variant CYP1B1 proteins could be different. It was interesting to note that 11 out of 23 mutants had lower stability relative to WT CYP1B1. This observation is not surprising since all 11 variants produced nonconservative missense mutations with side chains greatly dissimilar from WT. While the unstable nature of the seven mutant proteins was possibly the cause of their lower enzymatic activity, this observation did not appear to hold for the remaining four mutants. In the case of these four mutants, the rate of translation increased slowly and steadily up to the maximum period of tracking (46h), but appeared to be less stable when the translation was arrested at an early time point with CHX due to a lower accumulation of protein. This observation was consistent with the expression levels of these four mutants as measured from the lysates of cells untreated with CHX (S5 Fig).

**Fig 8:**
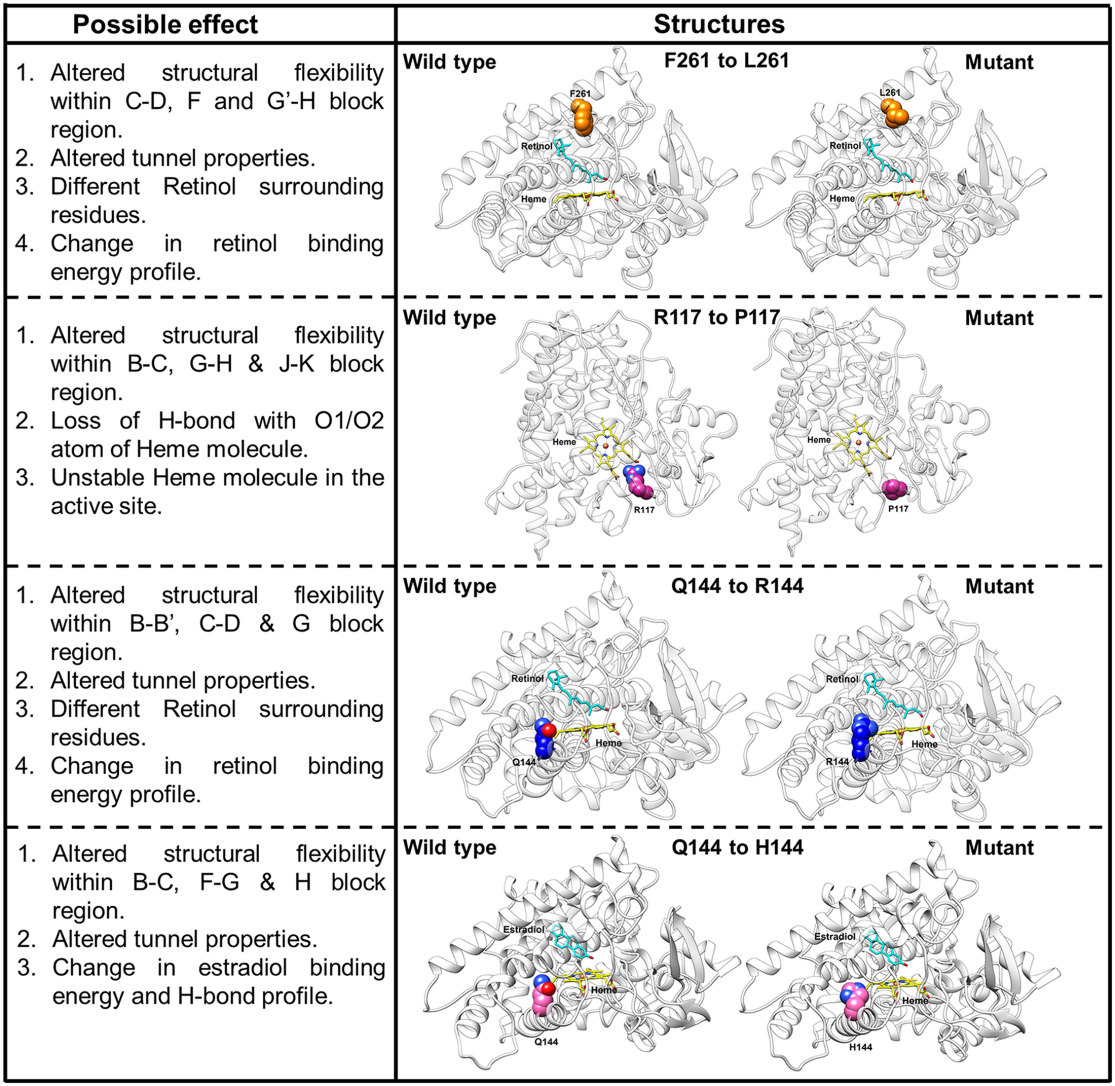
Summary of possible effects of mutations on CYP1B1 structure observed through MD simulation.

We performed extensive NMA, MD simulations and structural analyses to understand the probable structural alterations in the mutants of the CYP1B1 protein that led to widely varied functional results. We observed significant variation in the fluctuation of certain segmental parts of the mutant proteins with respect to their WT counterpart through NMA studies. The change in fluctuation within and surrounding the B-C and F-G loop region was previously known to affect the dynamic behavior and ligand entry/exit properties of the CYP1B1 family of proteins [36–39]. Our computational analyses showed that the relatively higher retinol metabolism activity of the F261L mutant could be a result of additional retinol-specific tunnels with lower path length and higher diameter, leading to improved retinol binding in F261L compared to the WT CYP1B1. Further, our structural analyses suggested that altered essential motions and fluctuations as a reflection of the R117P mutation might lead to compromised heme binding stability. Similarly, the null retinol biochemical activity of the Q144R mutation can also be attributed to the significantly changed motions of the crucial B-B’, C-D loop regions. Our study also indicated that the Q144R mutation might generate altered retinol-specific ligand tunnels, leading to compromised retinol entry/exit to the active site. On the other hand, the Q144H mutant shows lower 17β-estradiol metabolism activity and MD simulation analysis revealed that essential motions and fluctuation of the B-C, F-G and H block regions were significantly changed. The binding energy and hydrogen bonding profile (S9B Fig) of the Q144H mutant model suggested slightly decreased binding capability to 17β-estradiol. All of the results and possible effects of the tested mutations on the CYP1B1 structure are summarized in Fig 8.

It was observed that most of the PCG causing mutations in CYP1B1 are present as homozygous or compound heterozygous genotype whereas POAG causing mutations were heterozygous with very few exceptions. Thus, in most of the cases of PCG, we were able to relate the reported genotype with observed disease phenotype (Table 1). Additionally we could identify the cases where the genotype data are not consistent with our hypothesis based on the estimated RA metabolizing activity and speculate the reason.

Our results are also consistent with the observation that a mutation in a single allele of *CYP1B1* induces late onset glaucoma (i.e. POAG) due to the lower steroid metabolism of the mutant allele, as explained earlier. However, there are reports of a few exceptional cases where this genotype to phenotype correlation did not follow. For example the P52L, Y81N and E229K mutations were found in PCG patients in heterozygous conditions. It is interesting to note that close relatives of the PCG patients carrying the P52L and Y81N mutations with the same genotype were found to be unaffected [42, 58]. We predict that some unidentified genetic factors other than a mutation in *CYP1B1* might have role in PCG causation in these patients. Multiple reports of heterozygous E229K mutations in PCG cases suggest the involvement of an unidentified mutation in the other allele [5]. It is noteworthy that, the identical twin brother of a JOAG patient carrying a G61E [50] mutation had PCG. We assume that there might be other epigenetic and environmental factors that are responsible for controlling PCG onset in these cases provided there was no genotyping error.

Finally, our observations reveal that the mutations, that caused severe alteration in RA-metabolism without any comprehensive alteration in estradiol metabolism activity, were responsible solely for PCG pathogenesis (R117P and Q144R), whereas, the mutations that caused steroid metabolism dysfunction without any significant alteration in retinol metabolism were responsible for POAG (Q144H, V409F). However, Q144R has also been reported in the case of a single POAG patient [51]. We argue that this variant may not be associated with suspected POAG pathogenesis; the variant was found to have 17β-estradiol metabolism activity similar to the WT allele. Thus, this case further lends support to the argument that the association of a genomic variant with a phenotype should be examined by alternation of relevant biological function of the mutant molecule. The cases of mutations reported for both PCG and POAG also showed dysfunction in both the enzymatic activities. This may imply that if these mutations are present in heterozygous condition, the carrier of the mutation is able to acquire the threshold level of RA and escapes PCG but the onset of POAG takes place due to a reduction of steroid metabolism at a later age. Our hypothesis is supported by the reported occurrence of two glaucoma patients in a family: (i) a PCG patient carrying F261L and R355fsX69 in CYP1B1, and (ii) an ocular hypertensic patient carrying a single mutation (F261L) in CYP1B1 [42]. A more recent study by Lopez Garido et al [96] showed the coexistence of POAG and PCG phenotypes in the same family. The grandmother of the proband, heterozygous for a CYP1B1 mutation was detected with POAG at the age of 70, where as the proband with the same mutation in compound heterozygous condition along with a frame-shift mutation manifested PCG, strongly supporting our hypothesis.

Our study is highly suggestive of dual effect of *CYP1B1* in the families harboring mutation in the gene. We propose that the heterozygous parents of a PCG-affected child due presence of two defective alleles of *CYP1B1* are prone to late onset glaucoma due to altered metabolizing activity of 17β-estradiol relative to the normal levels. This hypothesis could directly be tested in the *CYP1B1* mediated PCG family by a study design that include long term follow up of the members of the families harboring the mutant *CYP1B1* allele in heterozygous condition. In the current study, we investigate the effect of several disease causing mutations in CYP1B1 on the biological activity of the protein. In many cases we were able to relate the measured enzymatic activities of the CYP1B1 mutants with disease pathogenesis, suggesting that functional analysis of any suspect variants in CYP1B1 could be predictive of disease pathogenesis, depending on the specific genotype at the given locus.

## Supporting Information

S1 Fig Endogenous expression of CYP1B1 in Trabecular Meshwork (TM) and HEK293T cell line. Each lane contains cell lysate corresponding to 20μg of total protein. CYP1B1 were detected by immunoblot using a monoclonal anti-CYP1B1-antibody (Santa Cruz Inc, USA). β-actin was detected using an anti-β-actin-antibody (Sigma, USA).
(TIF)

S2 Fig *CYP1B1* gene showing its different domain with naturally occurring missense mutations on it. Panel A illustrates mutations distributed on *CYP1B1* gene showing lack of any disease specific clustering of mutations. Panel B shows mutations selected for this study and the associated disease phenotypes are indicated by color code mentioned in the figure. Panel C shows the log transformed fluctuation ratio of the CYP1B1 mutants to CYP1B1 Wild Type structure. The fluctuations were obtained through normal mode analysis method. Panel **i** (left upper two panels) shows the fluctuation ratio of only PCG causing CYP1B1 mutants. F261L and R117P showed the highest extent of altered flexibility within the same B-C and F-G block region. Panel **ii** (lower left panel) shows the fluctuation ratio of only POAG causing CYP1B1 mutants but no specific altered flexibility is observed within the structure. Panel **iii** (right panels) displays both PCG and POAG causing CYP1B1 mutants. Q144R shows the highest extent of altered flexibility as compared to other known PCG and POAG causing mutants.
(TIF)

S3 Fig The protein levels of different CYP1B1 mutants present in transiently transfected HEK293T cells are shown at the time of estradiol metabolism (46h) assay in *Panel A* and at the time of retinol metabolism assay (36h) in *Panel B*. Each lane contains cell lysate corresponding to 20μg of total protein. CYP1B1 polypeptides tagged with myc-epitope at their C-terminal end were detected by immunoblot using a monoclonal anti-myc-antibody. β-actin was detected using an anti-β-actin-antibody.
(TIF)

S4 Fig Representative graph & western blots showing stability of three CYP1B1 variants at 0 min, 15 min, 30 min, 1h, 2h, 3h and 4h after inhibition of translation by CHX treatment. These mutants showed very little or no expression at 4 hours of treatment.
(TIF)

S5 Fig Percent expression of mutants (with respect to WT) at 14h, 18h, 22h and 26h without addition of CHX. (TIF)

S6 FigEuclidean distance of the Principal Components shows the relative similarity of each mutant with respect to the wild type CYP1B1 structure.The Euclidean distance among the three principal components (PC) can be calculated as follows:
$Euclidean\;distance\;=\;\sqrt{{\left({PC1}_{Wild\;type}-{PC1}_{Mutant}\right)}^{2}+{\left({PC2}_{Wild\;type}-{PC2}_{Mutant}\right)}^{2}+{\left({PC3}_{Wild\;type}-{PC3}_{Muatnt}\right)}^{2}}$(TIF)

S7 Fig Panel A shows that F261L mutant has a significantly altered flexibility pattern within the C-D, F and G’-H block region. Panel B shows the tunnel surrounding residues (≤5Å radius) in both the mutant and wild type structures. Panels C and D describe the binding energy profile and the surrounding residues of retinol within the F261L and wild type structures.
(TIF)

S8 Fig Panel A shows that R117P mutant possesses a significant altered flexibility pattern within the B-C, G-H, and J-K block regions. Panel B shows the contact analysis of heme O1A/O2A atoms within its surrounding ≤3.5Å radius.
(TIF)

S9 Fig Panel A shows that Q144R mutant has a significantly altered flexibility pattern within the B-B’, C-D, and G block region. Panel B shows the tunnel surrounding residues (≤ 5Å radius) in both the mutant and wild type structures. Panels C and D describe the binding energy profile and the surrounding residues of retinol within the Q144R and wild type structure.
(TIF)

S10 Fig Panel A shows that Q144H mutant possesses a significant altered flexibility pattern within the B-C, F-G and H block regions. Panels C and D describe the binding energy profile and the number of hydrogen bonds between estradiol and surrounding residues within the Q144H and wild type structure.
(TIF)

S1 Method Detailed Methodology of Molecular modelling and Docking analysis and Essential dynamics and Principal Component analysis have been provided with relevant references. (DOCX)

S1 Table List of subclones with their association with different diseases & primer sequences for site directed mutagenesis. (DOCX)

S2 Table Optimization of concentration of CHX on WT at different time points. (DOCX)

S3 Table The available assay results for the second mutations of compound heterozygous genotypes related to Table 1. (DOCX)
