# Supplementary figures and images for: Functional and Structural Analyses of CYP1B1 Variants Linked to Congenital and Adult-Onset Glaucoma to Investigate the Molecular Basis of These Diseases
Source: PLoS One. 2016 May 31;11(5):e0156252. doi: 10.1371/journal.pone.0156252 (PMC4887111; doi:10.1371/journal.pone.0156252)

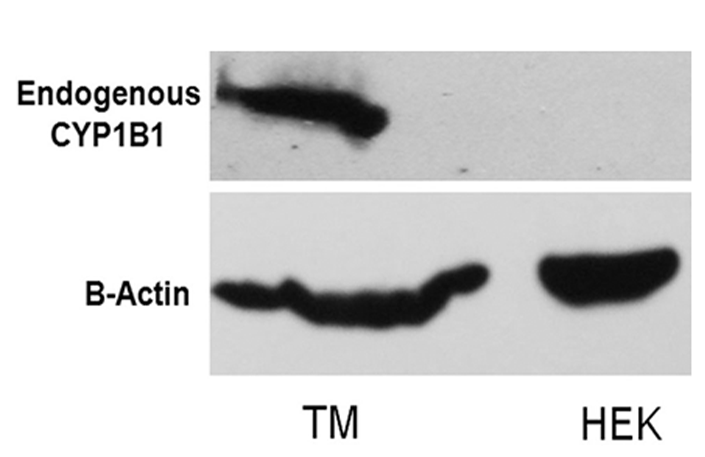

Supplement: S1 Fig — Each lane contains cell lysate corresponding to 20μg of total protein. CYP1B1 were detected by immunoblot using a monoclonal anti-CYP1B1-antibody (Santa Cruz Inc, USA). β-actin was detected using an anti-β-actin-antibody (Sigma, USA). (TIF) [file pone.0156252.s001.tif]

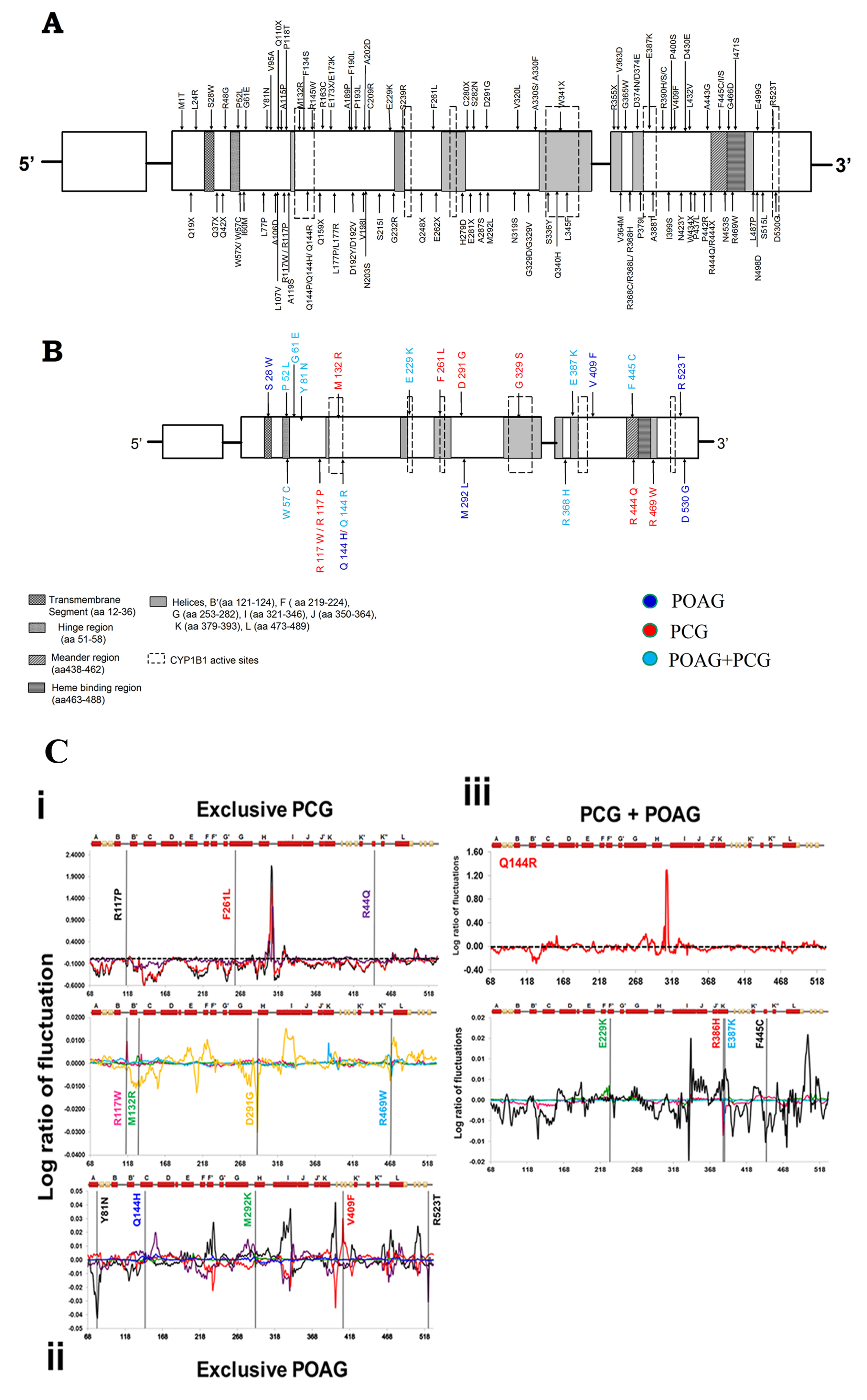

Supplement: S2 Fig — Panel A illustrates mutations distributed on CYP1B1 gene showing lack of any disease specific clustering of mutations. Panel B shows mutations selected for this study and the associated disease phenotypes are indicated by color code mentioned in the figure. Panel C shows the log transformed fluctuation ratio of the CYP1B1 mutants to CYP1B1 Wild Type structure. The fluctuations were obtained through normal mode analysis method. Panel i (left upper two panels) shows the fluctuation ratio of only PCG causing CYP1B1 mutants. F261L and R117P showed the highest extent of altered flexibility within the same B-C and F-G block region. Panel ii (lower left panel) shows the fluctuation ratio of only POAG causing CYP1B1 mutants but no specific altered flexibility is observed within the structure. Panel iii (right panels) displays both PCG and POAG causing CYP1B1 mutants. Q144R shows the highest extent of altered flexibility as compared to other known PCG and POAG causing mutants. (TIF) [file pone.0156252.s002.tif]

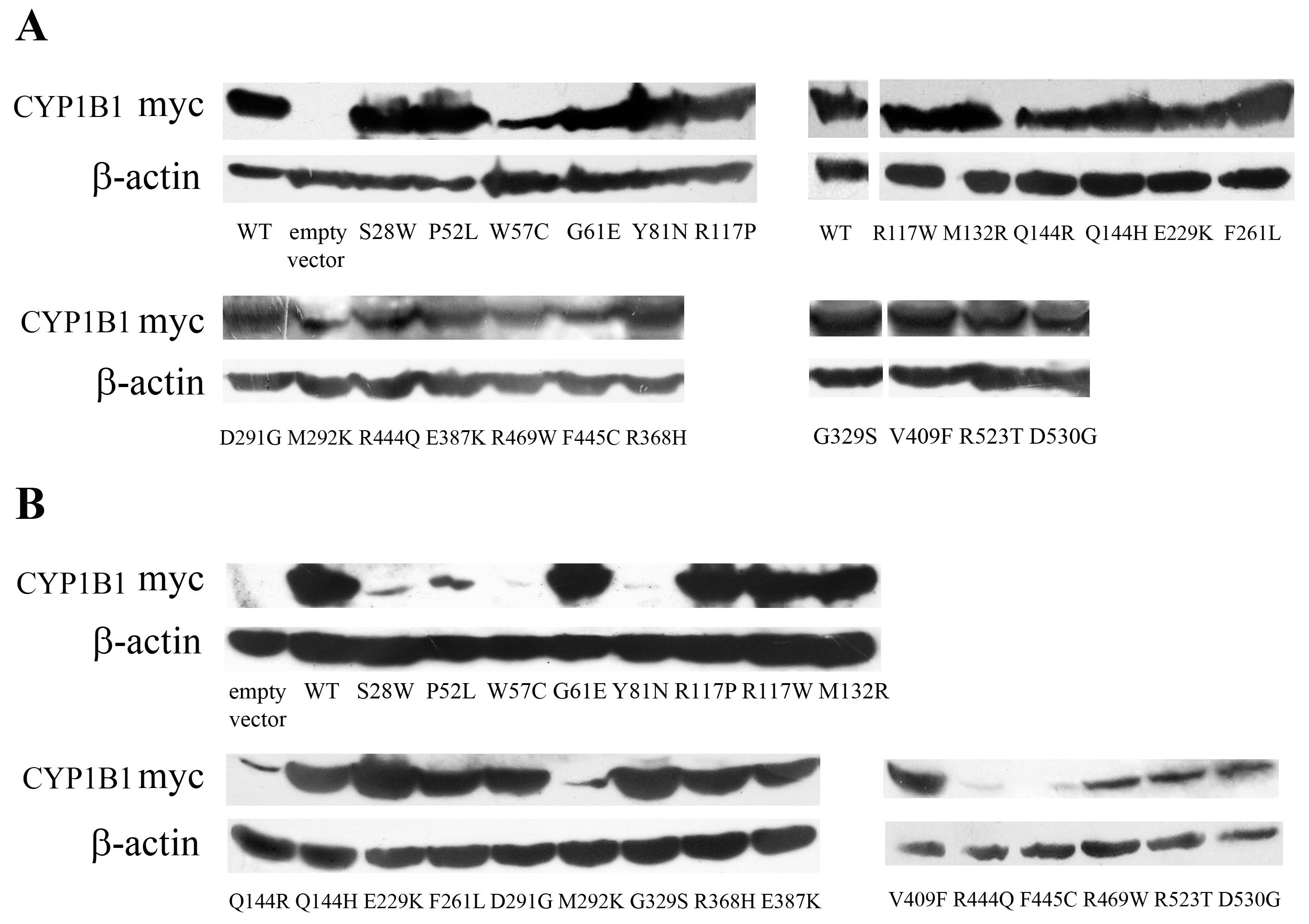

Supplement: S3 Fig — Each lane contains cell lysate corresponding to 20μg of total protein. CYP1B1 polypeptides tagged with myc-epitope at their C-terminal end were detected by immunoblot using a monoclonal anti-myc-antibody. β-actin was detected using an anti-β-actin-antibody. (TIF) [file pone.0156252.s003.tif]

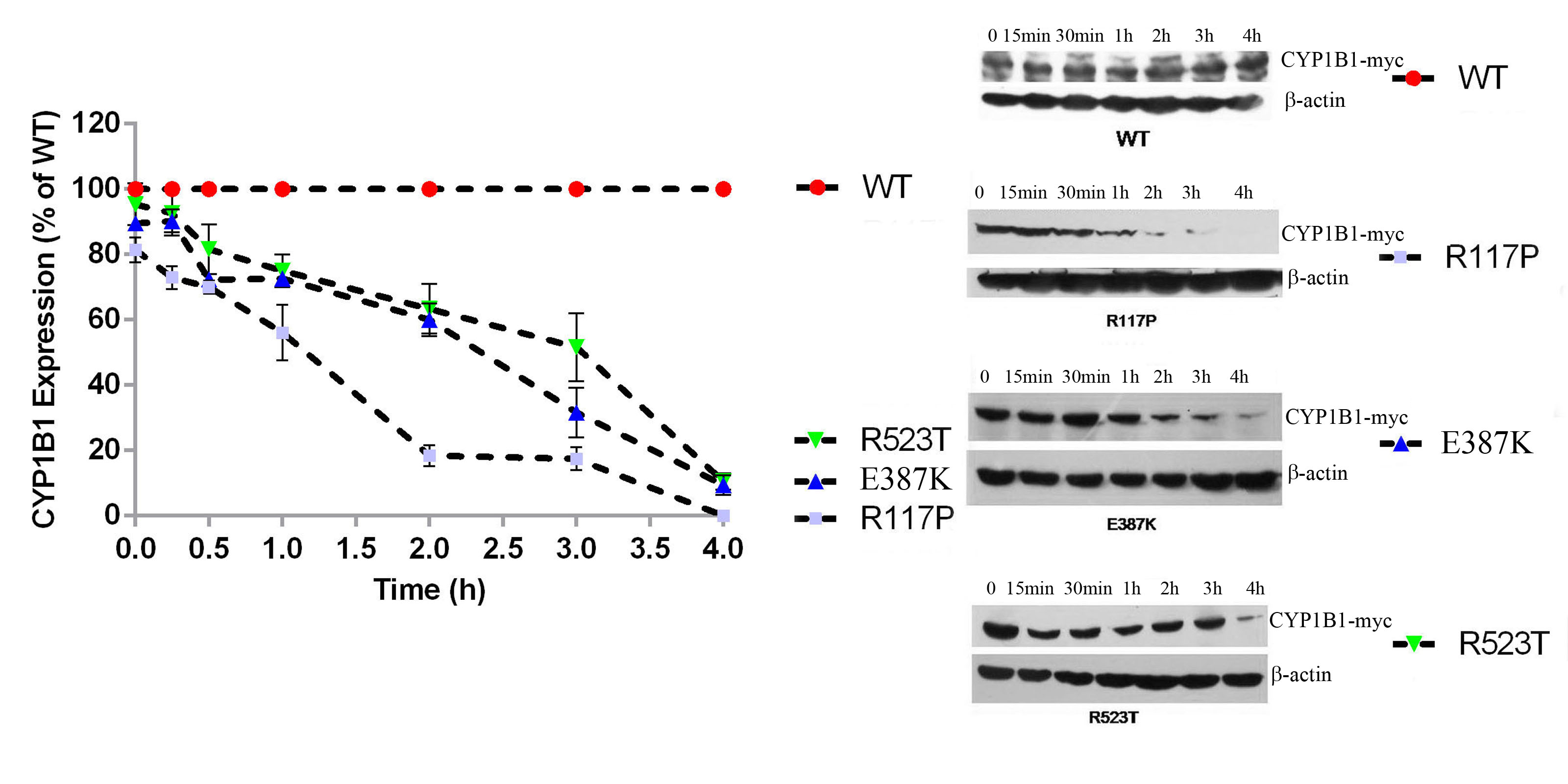

Supplement: S4 Fig — These mutants showed very little or no expression at 4 hours of treatment. (TIF) [file pone.0156252.s004.tif]

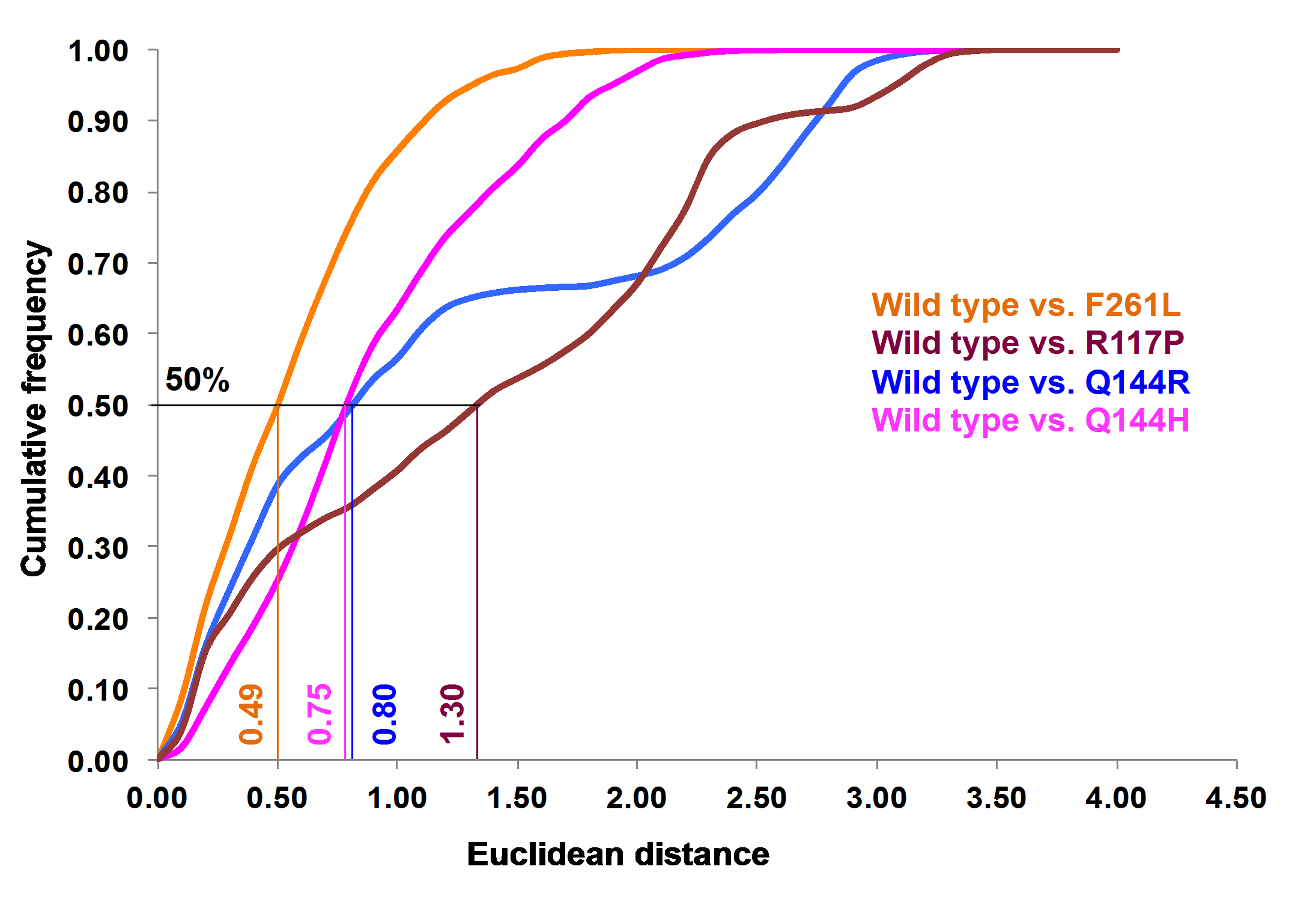

Supplement: S6 Fig — The Euclidean distance among the three principal components (PC) can be calculated as follows: Euclidean distance = (PC1Wild type-PC1Mutant)2+(PC2Wild type-PC2Mutant)2+(PC3Wild type-PC3Muatnt)2 (TIF) [file pone.0156252.s006.tif]

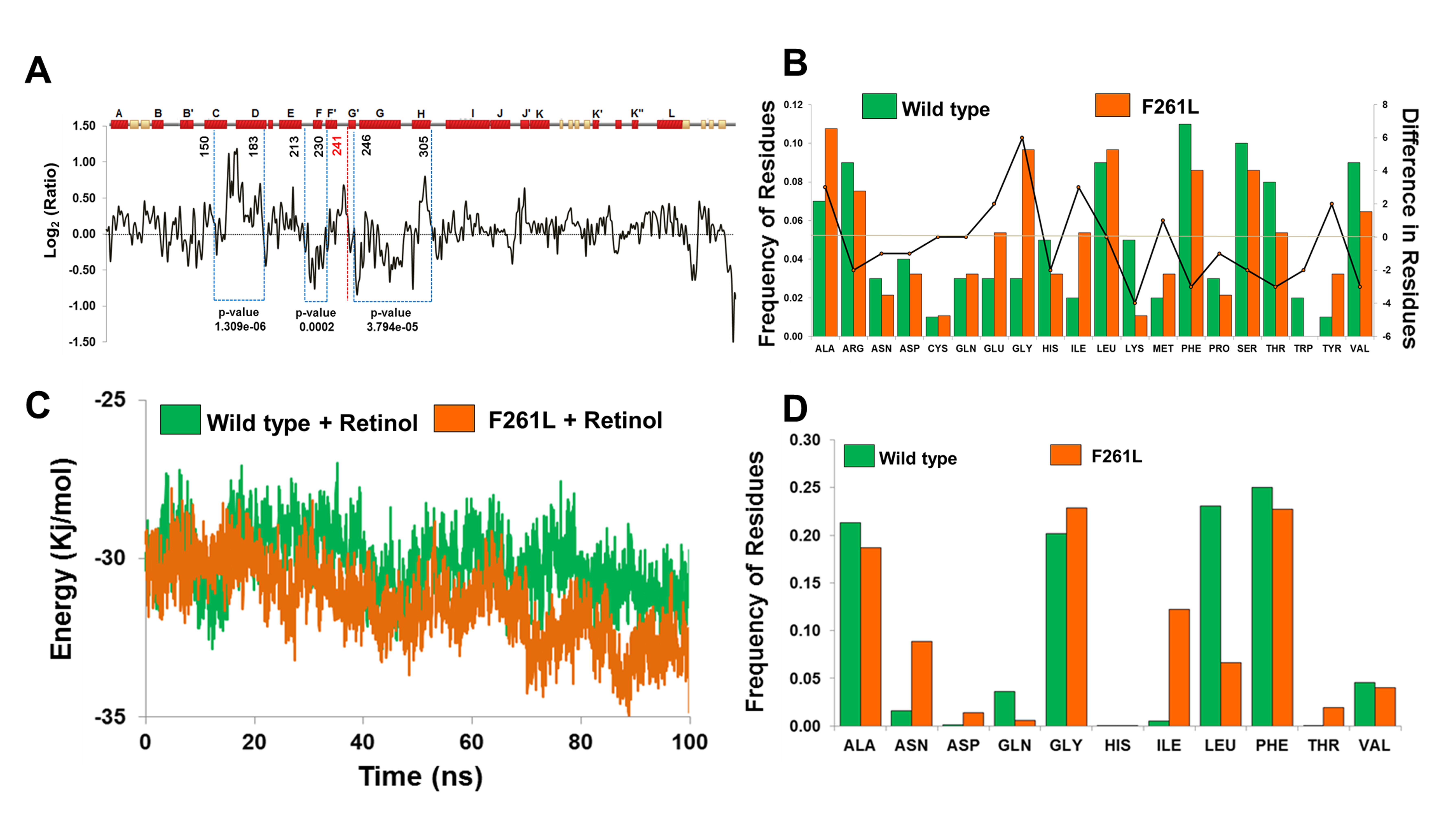

Supplement: S7 Fig — Panel A shows that F261L mutant has a significantly altered flexibility pattern within the C-D, F and G’-H block region. Panel B shows the tunnel surrounding residues (≤5Å radius) in both the mutant and wild type structures. Panels C and D describe the binding energy profile and the surrounding residues of retinol within the F261L and wild type structures. (TIF) [file pone.0156252.s007.tif]

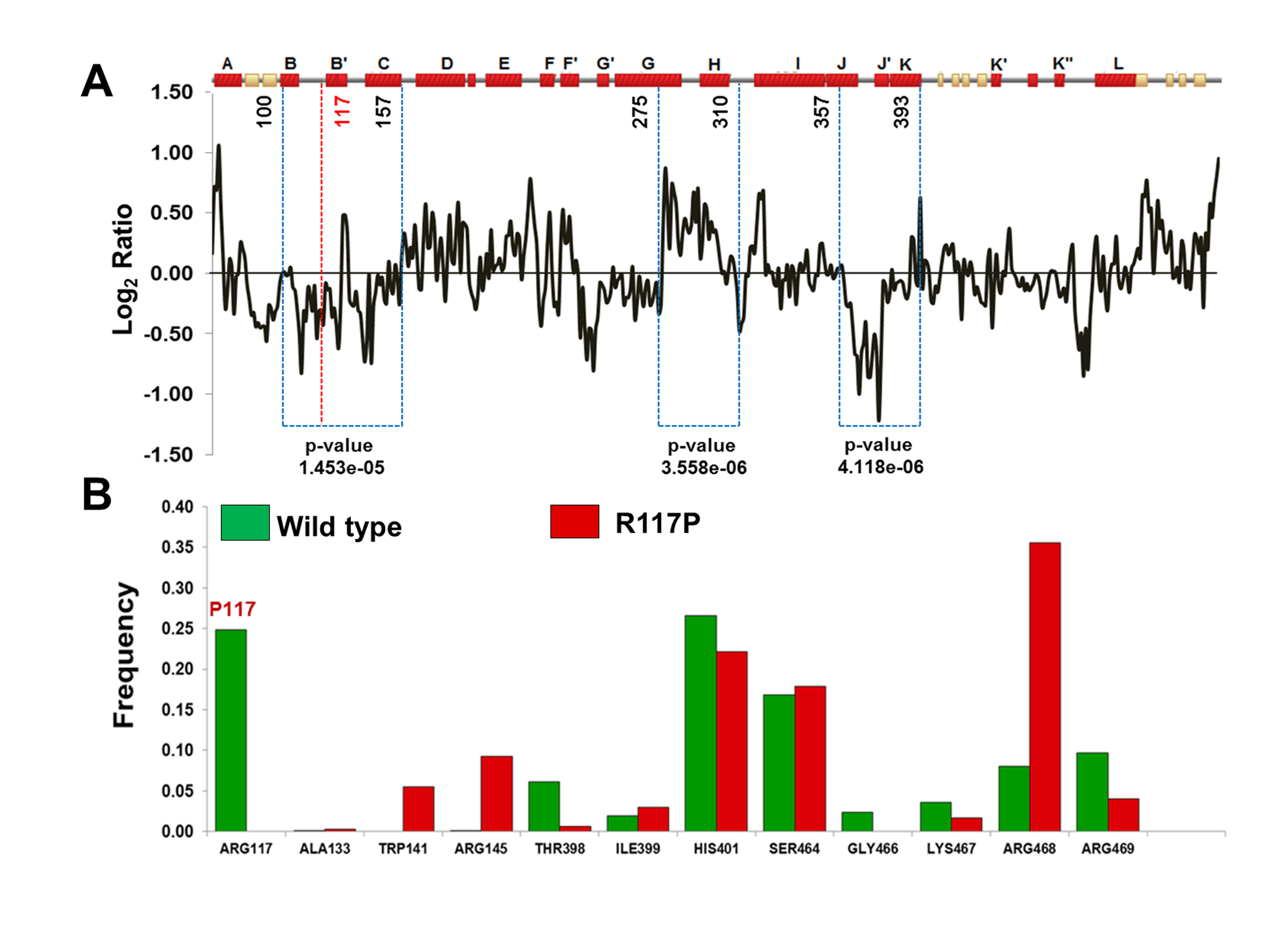

Supplement: S8 Fig — Panel A shows that R117P mutant possesses a significant altered flexibility pattern within the B-C, G-H, and J-K block regions. Panel B shows the contact analysis of heme O1A/O2A atoms within its surrounding ≤3.5Å radius. (TIF) [file pone.0156252.s008.tif]

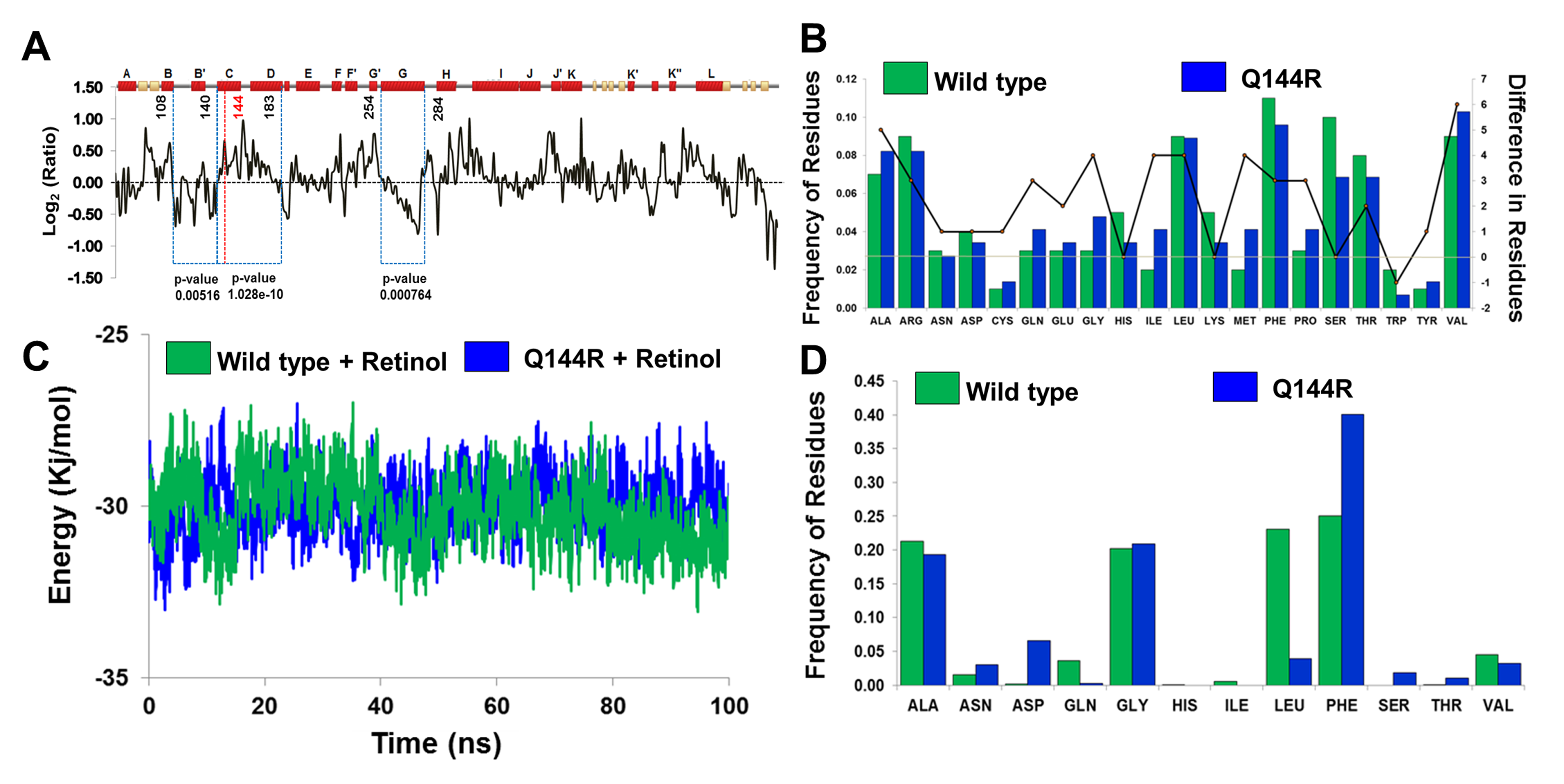

Supplement: S9 Fig — Panel A shows that Q144R mutant has a significantly altered flexibility pattern within the B-B’, C-D, and G block region. Panel B shows the tunnel surrounding residues (≤ 5Å radius) in both the mutant and wild type structures. Panels C and D describe the binding energy profile and the surrounding residues of retinol within the Q144R and wild type structure. (TIF) [file pone.0156252.s009.tif]

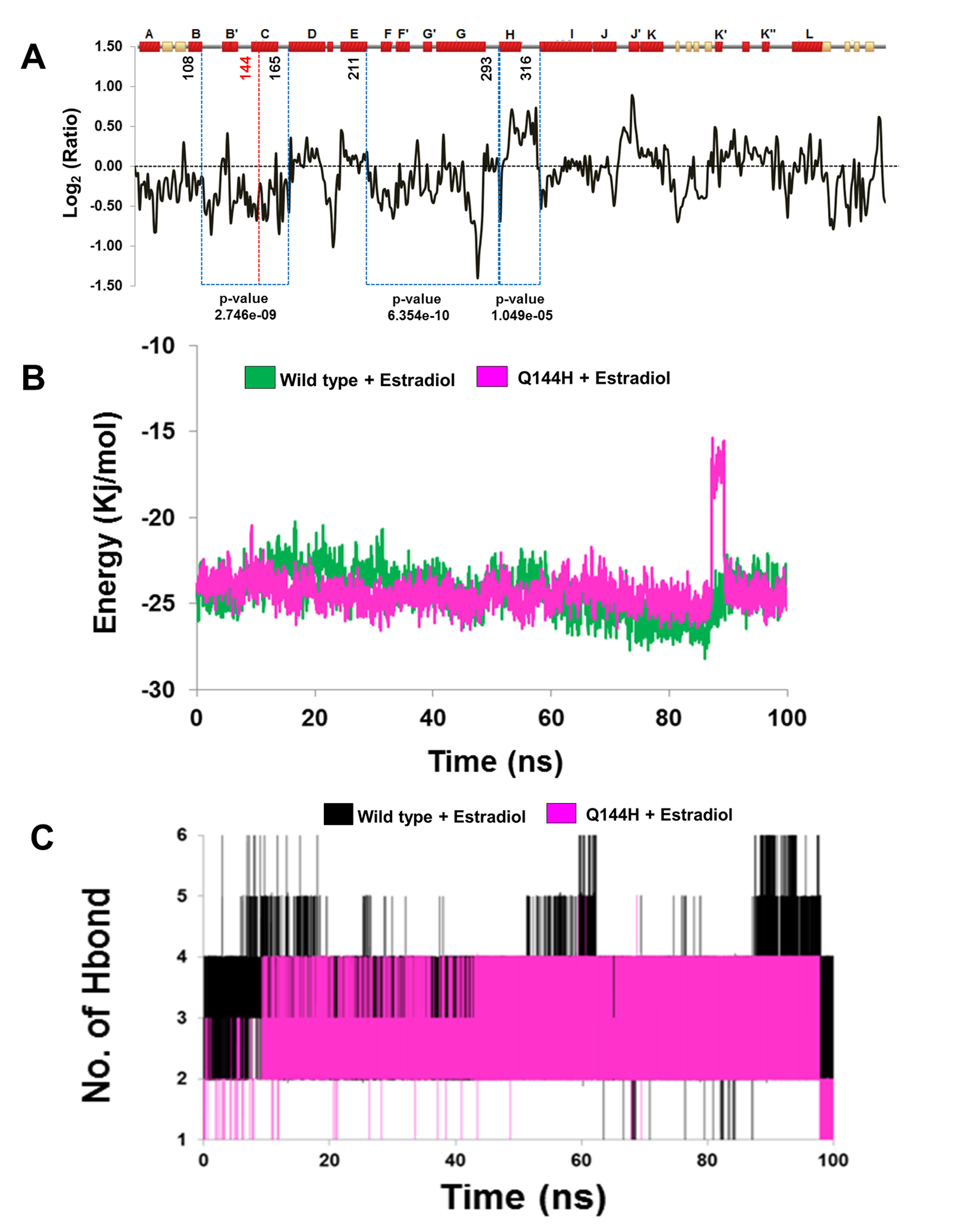

Supplement: S10 Fig — Panel A shows that Q144H mutant possesses a significant altered flexibility pattern within the B-C, F-G and H block regions. Panels C and D describe the binding energy profile and the number of hydrogen bonds between estradiol and surrounding residues within the Q144H and wild type structure. (TIF) [file pone.0156252.s010.tif]
