# Supplementary material for: Functional and Structural Analyses of CYP1B1 Variants Linked to Congenital and Adult-Onset Glaucoma to Investigate the Molecular Basis of These Diseases
Source: PLoS One. 2016 May 31;11(5):e0156252. doi: 10.1371/journal.pone.0156252 (PMC4887111; doi:10.1371/journal.pone.0156252)
Supplement: S1 Method — (DOCX) [file pone.0156252.s011.docx]

**S1 Method**

**Molecular modelling and docking analysis**

Molecular docking of retinol and 17β-estradiol ligands onto the CYP1B1 wild type and mutant structures were performed using the GOLD v5.0.1 [1] package from Cambridge Crystallographic Data centre. The 3D coordinates of retinol and 17β-estradiol were collected from the PubChem database. GOLD [1] software optimizes the fitness score of many possible docking solutions using a genetic algorithm. Following parameters were used in the docking cycles: population size (100), selection pressure (1.100000), number of operations (100,000), number of islands, niche size, crossover weight, mutate weight, and migrate weight. 100 docking calculations were run for each ligand and the best docking solutions were identified based on critical manual inspection satisfying favorable interactions between the ligand and the protein molecule. *GOLD score and Clash penalty scores* from the GOLD package were further used to identify the lowest energy-docking and most favorable modes among the manually selected docking solutions.

**Essential dynamics and principal component analysis**

Molecular Dynamics (MD) simulations allow us to study the structural motions of proteins in great detail. To obtain a concise interpretation of the protein motions from the large amount of simulation data a variety of dimensionality reduction methods are used [2-5]. These dimensionality reduction methods reduce the description of correlated molecular motions of 3N atomic coordinates to few collective degrees motions also known as the essential dynamics of the system. A widely used approach in this category is the principal component analysis (PCA). PCA approach diagonalizes the mass-weighted covariance matrix of 3N atomic coordinates obtained from simulation and thus removes the linear correlations among the atomic coordinates [6]. This linear transformation of collective motions further can be described by the ordering of eigenvectors or principal components decreasingly and it is been shown that a system's motions can be described by first few principal components [7].

A PCA or essential dynamics analysis of a protein MD simulation is carried out in the following three steps [7]:

Step1: The individual configurations of the ensemble obtained from the simulation are superimposed to the reference structure. In our case, this reference structure represents the wild type CYP1B1 crystal structure.

Step2: The superimposed trajectory is then used to build an atomic mass-weighted covariance-matrix which represents the variance of motions for each pair of atomic coordinates i.e. a 3N X 3N symmetric matrix. Atom pairs, which move in a correlated manner give rise to positive covariances while those move in anti-correlated manner produce negative covariances. Zero or near-zero covariances represent non-correlated atomic motions. The mass-weighted covariance matrix is then diagonalized to get a set of eigenvalues and eigenvectors. Eigenvalues represent the variance in the system's motions corresponding to collective modes/components or eigenvectors representing the collective motions of the system.

Step3: The ensemble is then projected onto each of the principal modes or components to describe the distribution of the protein conformations in their individual principal coordinates.

Therefore, these individual principal components of each structural conformation represent a set of collective motions exhibited by the 3D structure. Principal components are sorted decreasingly based on their relative weight in representing the collective motions, e.g. principal component 1 or pc1 will always represent the largest set of collective motions of the system followed by pc2 and pc3. Again all the principal components have their own scale of coordinate in the Euclidean space where the distribution of ensemble are represented and as both the normal and mutant CYP1B1 conformations are superimposed onto the same CYP1B1 crystal structure to yield the principal components the respective principal component values are considered to be normalized and unit less.

**References**

1. Verdonk ML, Cole JC, Hartshorn MJ, Murray CW, Taylor RD. Improved protein-

ligand docking using GOLD. Proteins. 2003;52:609-623.

2. Rohrdanz MA, Zheng W, Clementi C. Discovering mountain passes via torchlight: methods for the definition of reaction coordinates and pathways in complex macromolecular reactions. Annu Rev Phys Chem. 2013;64:295-316.

3. Das P, Moll M, Stamati H, Kavraki LE, Clementi C. Low-dimensional, free-energy landscapes of protein-folding reactions by nonlinear dimensionality reduction. Proc Natl Acad Sci U S A. 2006;103(26):9885-90

4. Krivov SV, Karplus M. Diffusive reaction dynamics on invariant free energy profiles. Proc Natl Acad Sci U S A. 2008;105(37):13841-6.

5. Krivov SV. On Reaction Coordinate Optimality. J Chem Theory Comput. 2013;9(1):135-46. doi: 10.1021/ct3008292. PubMed PMID: 26589017.

6. Sittel F, Jain A, Stock G. Principal component analysis of molecular dynamics: on the use of Cartesian vs. internal coordinates. J Chem Phys. 2014;141(1):014111.

7. Amadei A, Linssen AB, Berendsen HJ. Essential dynamics of proteins. Proteins. 1993:17: 412-425.
