# Supplementary material for: Functional and Structural Analyses of CYP1B1 Variants Linked to Congenital and Adult-Onset Glaucoma to Investigate the Molecular Basis of These Diseases
Source: PLoS One. 2016 May 31;11(5):e0156252. doi: 10.1371/journal.pone.0156252 (PMC4887111; doi:10.1371/journal.pone.0156252)
Supplement: S1 Table — (DOCX) [file pone.0156252.s012.docx]

**S1 Table**

| **MUTATION** | **ASSOCIATION WITH DISEASE** | **PRIMER SEQUENCES** |
| --- | --- | --- |
| Ser 28 Trp | POAG | F- CCTGCTACTCCTGTGGGTGCTGGCCACTGTG |
|  |  | R- CACAGTGGCCAGCACCCACAGGAGTAGCAGG |
| Arg48Gly | PCG, POAG, Breast Cancer | F- GGAGGCGGCAGCTCGGGTCCGCGCCCCCGG |
|  |  | R- CCGGGGGCGCGGACCCGAGCTGCCGCCTCC |
| Trp57Cys | PCG, POAG | F- GGCCCGTTTGCGTGCCCACTGATCGGAAACGC |
|  |  | R- GCGTTTCCGATCAGTGGGCACGCAAACGGGCC |
| Gly 61 Glu | PCG | F-GTTTGCGTGGCCACTGATCGAAAACGCGGCGGCGGTG |
|  |  | R-CACCGCCGCCGCGTTTTCGATCAGTGGCCACGCAAAC |
| Tyr 81 Asn | POAG | F-CCTGGCGCGGCGCAACGGCGACGTTTTCC |
|  |  | R-GGAAAACGTCGCCGTTGCGCCGCGCCAGG |
| Arg 117 Pro | PCG | F-CGGCCTTCGCCGACCCGCCGGCCTTCGCC |
|  |  | R-GGCGAAGGCCGGCGGGTCGGCGAAGGCCG |
| Arg117Trp | PCG | F- CGGCCTTCGCCGACTGGCCGGCCTTCGCC |
|  |  | R- GGCGAAGGCCGGCCAGTCGGCGAAGGCCG |
| Gln144Arg | PCG | F- CACTGGAAGGTGCGGCGGCGCGCAGCCCAC |
|  |  | R- GTGGGCTGCGCGCCGCCGCACCTTCCAGTG |
| Gln144His | POAG | F- CACTGGAAGGTGCATCGGCGCGCAGCCCAC |
|  |  | R- GTGGGCTGCGCGCCGATGCACCTTCCAGTG |

| **MUTATION** | **ASSOCIATION WITH DISEASE** | **PRIMER SEQUENCES** |
| --- | --- | --- |
| Glu 229 Lys | PCG, Cancer | F-GAGCTGCTCAGCCACAACAAAGAGTTCGGGCGCACG |
|  |  | R-CGTGCGCCCGAACTCTTTGTTGTGGCTGAGCAGCTC |
| Phe261Leu | PCG | F- CCGTTTTCCGCGAATTAGAGCAGCTCAACCGC |
|  |  | R- GCGGTTGAGCTGCTCTAATTCGCGGAAAACGG |
| Asp291Gly | PCG | F- GCCGCCCCCCGCGGCATGATGGACGCC |
|  |  | R- GGCGTCCATCATGCCGCGGGGGGCGGC |
| Met292Lys | POAG | F- GGGCCGCCCCCCGCGACAAGATGGACGCCTTTATCC |
|  |  | R- GGATAAAGGCGTCCATCTTGTCGCGGGGGGCGGCCC |
| Gly 329 Ser | POAG | F-CTATCACTGACATCTTCAGCGCCAGCCAGGACACCC |
|  |  | R-GGGTGTCCTGGCTGGCGCTGAAGATGTCAGTGATAG |
| Arg368His | PCG, POAG | F- GGTCGTGGGGAGGGACCATCTGCCTTGTATGGGTG |
|  |  | R- CACCCATACAAGGCAGATGGTCCCTCCCCACGACC |
| Glu 387 Lys | JOAG, PCG | F-CCTATGTCCTGGCCTTCCTTTATAAAGCCATGCGCTTCTCCAGC |
|  |  | R-GCTGGAGAAGCGCATGGCTTTATAAAGGAAGGCCAGGACATAGG |
| Val 409 Phe | POAG | F-CACTGCCAACACCTCTTTCTTGGGCTACCAC |
|  |  | R-GTGGTAGCCCAAGAAAGAGGTGTTGGCAGTG |
| Pro442Arg | PCG | F- CCCGGAGAACTTTGATCGAGCTCGATTCTTGGACAAGG |
|  |  | R- CCTTGTCCAAGAATCGAGCTCGATCAAAGTTCTCCGGG |
| Arg444Gln | PCG | F- GGAGAACTTTGATCCAGCTCAATTCTTGGACAAGGATGGC |
|  |  | R- GCCATCCTTGTCCAAGAATTGAGCTGGATCAAAGTTCTCC |
| Phe445Cys | POAG | F- CTTTGATCCAGCTCGATGCTTGGACAAGGATGGCC |
|  |  | R- GGCCATCCTTGTCCAAGCATCGAGCTGGATCAAAG |

| **MUTATION** | **ASSOCIATION WITH DISEASE** | **PRIMER SEQUENCES** |
| --- | --- | --- |
| Arg 469 Trp | PCG | F-CAGTGGGCAAAAGGTGGTGCATTGGCGAAG |
|  |  | R-CTTCGCCAATGCACCACCTTTTGCCCACTG |
| Arg 523 Thr | POAG | F-GTCAATGTCACTCTCACAGAGTCCATGGAGCTCC |
|  |  | R-GGAGCTCCATGGACTCTGTGAGAGTGACATTGAC |
| Asp 530 Gly | POAG | F-GTCCATGGAGCTCCTTGGTAGTGCTGTCCAAAATTTAC |
|  |  | R-GTAAATTTTGGACAGCACTACCAAGGAGCTCCATGGAC |
