# Supplementary material for: Functional and Structural Analyses of CYP1B1 Variants Linked to Congenital and Adult-Onset Glaucoma to Investigate the Molecular Basis of These Diseases
Source: PLoS One. 2016 May 31;11(5):e0156252. doi: 10.1371/journal.pone.0156252 (PMC4887111; doi:10.1371/journal.pone.0156252)
Supplement: S2 Table — (DOCX) [file pone.0156252.s013.docx]

**S2 Table: Optimization of concentration of CHX on WT at different time points.**

| **Conc. of Cycloheximide (μg/ml)** | **% of expression with respect to 14hr at 14 hr** | **% of expression with respect to 14hr at 18 hr** | **% of expression with respect to 14hr at 22 hr** | **% of expression with respect to 14hr at 26 hr** |
| --- | --- | --- | --- | --- |
| 5 | 100.00 ± 0.00 | 102.72 ± 1.50 | 158.68 ± 2.03 | 192.53 ± 2.39 |
| 10 | 100.00 ± 0.00 | 110.44 ± 1.60 | 100.54 ± 1.58 | 97.38 ± 2.11 |
| 15 | 100.00 ± 0.00 | 107.94 ± 1.36 | 97.71 ± 1.96 | 74.41 ± 2.53 |
