# Supplementary material for: Functional and Structural Analyses of CYP1B1 Variants Linked to Congenital and Adult-Onset Glaucoma to Investigate the Molecular Basis of These Diseases
Source: PLoS One. 2016 May 31;11(5):e0156252. doi: 10.1371/journal.pone.0156252 (PMC4887111; doi:10.1371/journal.pone.0156252)
Supplement: S3 Table — (DOCX) [file pone.0156252.s014.docx]

**S3 Table**

Available 2^nd^ mutation activity:

| **Mutation** | **Retinol Metabolism (% of WT)** | **Steroid Metabolism (% of WT)** |
| --- | --- | --- |
| Y81N | 0.00 ± 0.00 | 10.41 ± 5.50 |
| R469W | 124.20 ±25.30 | 5.79 ± 1.04 |
| M292K | 181.50 ± 6.84 | 0.00 |
| G61E | 0.00 ± 0.00 | 0.00 |
| R368H | 0.00 ± 0.00 | 21.29 ± 2.40 |
